# Unveiling potent inhibitors for schistosomiasis through ligand-based drug design, molecular docking, molecular dynamics simulations and pharmacokinetics predictions

**DOI:** 10.1371/journal.pone.0302390

**Published:** 2024-06-26

**Authors:** Saudatu Chinade Ja’afaru, Adamu Uzairu, Imren Bayil, Muhammed Sani Sallau, George Iloegbulam Ndukwe, Muhammad Tukur Ibrahim, Abu Tayab Moin, A. K. M. Moniruzzaman Mollah, Nurul Absar

**Affiliations:** 1 Department of Chemistry Ahmadu Bello University Zaria, Zaria, Nigeria; 2 Department of Chemistry, Aliko Dangote University of Science and Technology, Wudil, Kano, Nigeria; 3 Department of Bioinformatics and Computational Biology, Gaziantep University, Gaziantep, Turkey; 4 Department of Genetic Engineering and Biotechnology, Faculty of Biological Sciences, University of Chittagong, Chattogram, Bangladesh; 5 Department of Biological Sciences, Asian University for Women (AUW), Chattogram, Bangladesh; 6 Department of Biochemistry and Biotechnology, Faculty of Basic Medical and Pharmaceutical Sciences, University of Science & Technology Chittagong, Khulshi, Chittagong, Bangladesh; University of Nairobi, KENYA

## Abstract

Schistosomiasis is a neglected tropical disease which imposes a considerable and enduring impact on affected regions, leading to persistent morbidity, hindering child development, diminishing productivity, and imposing economic burdens. Due to the emergence of drug resistance and limited management options, there is need to develop additional effective inhibitors for schistosomiasis. In view of this, quantitative structure-activity relationship studies, molecular docking, molecular dynamics simulations, drug-likeness and pharmacokinetics predictions were applied to 39 *Schistosoma mansoni* Thioredoxin Glutathione Reductase (SmTGR) inhibitors. The chosen QSAR model demonstrated robust statistical parameters, including an R^2^ of 0.798, R^2^_adj_ of 0.767, Q^2^cv of 0.681, LOF of 0.930, R^2^_test_ of 0.776, and cR^2^p of 0.746, confirming its reliability. The most active derivative (compound **40**) was identified as a lead candidate for the development of new potential non-covalent inhibitors through ligand-based design. Subsequently, 12 novel compounds (**40a-40l**) were designed with enhanced anti-schistosomiasis activity and binding affinity. Molecular docking studies revealed strong and stable interactions, including hydrogen bonding, between the designed compounds and the target receptor. Molecular dynamics simulations over 100 nanoseconds and MM-PBSA free binding energy (ΔG_bind_) calculations validated the stability of the two best-designed molecules. Furthermore, drug-likeness and pharmacokinetics prediction analyses affirmed the potential of these designed compounds, suggesting their promise as innovative agents for the treatment of schistosomiasis.

## 1. Introduction

Schistosomiasis, a prevalent human parasitic infection, represents a significant global health challenge, impacting more than 200 million individuals in developing countries [1–4]. The disease is prevalent across sub-Saharan Africa, parts of South America, the Caribbean, the Middle East, and Southeast Asia [5]. Schistosomiasis exacts a heavy toll, causing approximately 280,000 deaths yearly [4]. Chronic infections of schistosomiasis can severely damage organs like the liver, spleen, and urinary tract and increase the risk of bladder cancer and infertility as reported by Silvestri V. and coworkers, along with many other researchers [6–8]. The predominant therapeutic approach for schistosomiasis relies on a single drug, Praziquantel (PZQ), which is administered extensively to combat the disease’s impact [9]. Despite its widespread use, PZQ’s effectiveness is compromised by several factors, including its exclusive activity against certain Schistosome species and the potential emergence of drug-resistant parasites [10–12]. Moreover, the absence of a reliable alternative to PZQ underscores a critical limitation in current treatment options. Hence, it is necessary to explore more potential inhibitors for Schistosomiasis.

The enzyme *Schistosoma mansoni* Thioredoxin Glutathione Reductase (SmTGR) plays a crucial role in the antioxidant defense system of the *Schistosoma* parasite, making it an attractive drug target for combatting schistosomiasis [13, 14]. SmTGR is involved in maintaining the redox balance within the parasite’s cells, enabling it to neutralize harmful reactive oxygen species (ROS) generated by the host’s immune system [15]. This function contributes to the parasite’s ability to evade immune attacks and establish infection [16]. Therefore, inhibiting it could disrupt the delicate redox balance that the parasite relies on for survival. According to the research conducted by Gustavo Salinas and colleagues, SmTGR exhibits structural and functional differences from its human counterparts, making it a potential target for selective inhibition [17]. Exploiting these differences could minimize the risk of adverse effects on the host. Also, in line with the discoveries of Jose T. Moreira-Filho and co-workers, as well as numerous other researchers, SmTGR is identified as a crucial survival mechanism for *Schistosomes*. Consequently, directing drugs at SmTGR has the potential to disrupt these vital processes, offering a promising approach for the development of antischistosomal medications [18, 19]. As a result, SmTGRs not only represent promising targets for drug development but also hold potential as candidates for the development of a vaccine against the parasite.

The traditional process of designing and developing drugs has been demanding, costly, and time-consuming [20]. Progress in computational science has revolutionized drug discovery, rendering it more efficient and economical [21–23]. The effective utilization of Computer-Aided Drug Design (CADD) signifies a notable advancement in drug discovery and development methodologies, offering a more cost-effective and efficient alternative to the conventional processes acknowledged for their prohibitive costs and time requirements. Recent progress in *in-silico* techniques has enabled the construction of physicochemical models to simulate biomolecular processes, empowering numerous research laboratories to innovate and discover novel medications [24]. Through CADD approaches, several potent and commonly prescribed drugs have been successfully developed to combat various life-threatening illnesses and pathogenic infections such as Human Immunodeficiency Virus (HIV), Influence Virus, Hepatitis C etc. notably example of such medications are Raltegravir (Isentress), Oseltamivir (Tamiflu), Zanamivir (Relenza), Boceprevir (Victrelis), Doravirine (Pifeltro) among others [25–29]. Various structure-based and/or ligand-based design methods are now employed, with ligand-focused techniques like Quantitative Structure-Activity Relationship (QSAR) gaining prominence [30, 31]. Robust QSAR models facilitate the economical virtual screening of extensive chemical databases, identifying potentially active compounds that meet the criteria for promising drug candidates.

In this research, we utilize ligand-based drug design (LBDD) as a method to discover potent non-covalent inhibitors of SmTGR with improved activity and enhanced binding capabilities. Non-covalent inhibitors of SmTGR offer several advantages over covalent inhibitors, including reversibility, selectivity, reduced potential for toxicity, ease of optimization, and lower risk of drug resistance. These properties make them promising candidates for the development of novel therapeutic agents against schistosomiasis [32]. Therefore, a collection of compounds exhibiting phenotypic antischistosomal activities served as the initial foundation for devising inhibitors targeting SmTGR, presenting a logical and effective method for identifying promising candidate compounds with possible therapeutic benefits against schistosomiasis [18]. This approach exploits on the already established efficacy of compounds against the parasite, enabling exploration of their molecular interactions and facilitating modification of drug candidates to enhance effectiveness and safety [33]. Consequently, the scope encompasses several computational techniques, including QSAR modeling, molecular docking, molecular dynamics (MD) simulations, drug-likeness assessment, and pharmacokinetics profiling. QSAR modeling will be employed to predict the activity of candidate compounds and guide the design of derivatives, while molecular docking simulations will identify potential binding sites and optimize inhibitor structures. MD simulations will explore the dynamic behavior of protein-ligand complexes under physiological conditions to refine inhibitor designs. Drug-likeness assessment and pharmacokinetics profiling will prioritize compounds with favorable pharmacokinetic properties. However, limitations exist, such as potential discrepancies between computational predictions and experimental results, the availability of accurate structural data, and resource and time constraints. The main goal of this study is to identify and characterize derivatives with potential application in the treatment of schistosomiasis using *in-silico* approach.

## 2. Materials and methods

### 2.1 Dataset collection, preparations, structure determination and optimization

The dataset was downloaded from ChEMBL (https://www.ebi.ac.uk/chembl) and included experimental data for Schistosoma mansoni as a target (target ID: CHEMBL6110). The dataset was screened and filtered to select compounds suitable for the QSAR study and was rigorously cleaned to eliminate duplicates and resolve discrepancies (**S1 Table**) [34]. Compounds with incomplete or inconsistent activity values were eliminated and data authenticity was verified to maintain data quality and integrity [35]. The biological activities, initially recorded as IC_50_ in nanomolar (nM), were transformed into pIC_50_ to achieve data linearity and uniformity throughout the dataset [36]. Following the filtration process, the dataset was reduced from the initial count of 57 compounds to 49, which were subsequently employed for further analysis.

The Simplified Molecular Input Line Entry System (SMILES) notation for each compound served as the foundation for constructing respective two-dimensional (2D) chemical structures using PerkinElmer ChemDraw software [37]. These structures were then transformed into a three-dimensional (3D) format utilizing Spartan v14.0 software. The optimization of molecular geometry was conducted on the Spartan interface through Density Functional Theory (DFT) quantum mechanical calculations employing the B3LYP/631-G* basis set [38]. The optimized geometric structures of the molecules were saved in a unified folder in Spatial Document File (sdf) format.

### 2.2 Descriptor calculations, data pretreatment and division

The Pharmaceutical Data Exploration Laboratory (PaDEL) descriptor toolkit was utilized to calculate essential molecular descriptors that contribute to the anti-schistosomiasis activities of the derivatives [39]. This involved importing the 3D structures saved in sdf file format into the PaDEL software. The chosen configuration included the selection of all descriptors (1D, 2D, and 3D), while for ’standardization’, various options were checked (remove salt, detect aromaticity, standardize tautomers, SMIRK tautomers file, standardize nitro groups and retain 3D coordinated) and the MM2 forcefield was employed [23]. Subsequently, the generated descriptors underwent manual preprocessing to eliminate redundant and highly correlated ones [40]. Further refinement was performed using version 1.2 of the pretreatment software. The dataset was then divided into training (modeling) and test (validation) sets using the Kennard-Stone algorithm [41]. The training set comprised 39 compounds, accounting for 80% of the dataset, while the remaining 10 compounds, constituting 20%, were set aside for the external validation test set [42].

### 2.3 Building and validation of QSAR Model

The training set compounds were employed for generating the QSAR model and performing internal validations, while the test set molecules were used for the model’s external validation and assessment of predictive performance [42]. Combination of the genetic function algorithm (GFA) with multi-linear regression (MLR) within Material Studio v8.0 was applied to generate the QSAR models [43]. Within the GFA regression, the biological activities (p1C_50_) served as the dependent variables, while the descriptor values were treated as independent variables. For ensuring model convergence, specific parameters were set: the population sample and maximum generation were fixed at 10,000 and 1500, respectively; the number of top equations returned was limited to 4; a mutation probability of 0.1 was employed, and the default smoothing parameter of 0.5 was maintained [43]. Identification and selection of the best QSAR model relied on key statistical parameters, encompassing the correlation coefficient of the training set (R^2^_internal_), adjusted correlation coefficient (R^2^_adj_), cross-validation coefficient (Q^2^cv), and correlation coefficient of the external test set (R^2^_external_) [44]. The equations characterizing these validation parameters are provided in Eqs (1–4), respectively.

$${R^{2}}_{internal}=1‐\frac{{\sum (Y_{exp}-Y_{pred})}^{2}}{\sum (Y_{exp}-{Ӯ}_{training}{)}^{2}}$$ (1)

$${R^{2}}_{adj}=\frac{R^{2}-C(B-1)}{B-C+1}$$ (2)

$$Q^{2}cv=1‐\frac{{\sum (Y_{pred}-Y_{exp})}^{2}}{\sum (Y_{exp}-{Ӯ}_{training}{)}^{2}}$$ (3)

$${R^{2}}_{external}=1‐\frac{{\sum (Y_{exp(test\;set)}-Y_{pred(test\;set)})}^{2}}{\sum (Y_{exp(test\;set)}-{Ӯ}_{training}{)}^{2}}$$ (4)

Where, $Y_{exp},Y_{pred}$ and ${Ӯ}_{training}$ are the experimental, predicted and average training set activities. B is the total no. of compounds used as training set and C is the no. of descriptors used to generate the model.

The chosen model underwent evaluation using the subsequent quantitative evaluations: mean effect (ME), variance inflation factor (VIF), and Y-scrambling analysis [22, 36, 45]. The ME measurement was employed to assess the significance of each descriptor’s role within the selected model, VIF was calculated to assess the multicollinearity between the descriptor’s while the Y-scrambling test was implemented to substantiate the model’s robustness [46]. The calculation of each descriptor’s ME was conducted utilizing Eq 5, which indicates the degree of influence exerted by the descriptors on the compounds’ activities.

The Y-scrambling test was performed by computing the coefficient of the validation parameter for Y-randomization (cR^2^p) using Eq 6. This process involved rearranging the actual activities while maintaining the descriptors unchanged. It was anticipated that the reshuffled QSAR model would exhibit low Q^2^ and R^2^ values, alongside a cR^2^p value surpassing 0.5, as an indicator of its reliability [47].

$$ME_{q}=\frac{A_{q}\sum_{q=1}^{w=n}dwq}{\sum_{q}^{m}A_{q}\sum_{w}^{n}d_{wq}}$$ (5)

Where, ME_q_ is the mean effect of descriptor q in the model, A_q_ is the coefficient of descriptor q of that model, and dwq is the value of descriptor q in the data matrix for each compound in the model building set. m is the sum of descriptors present in the model, and n is the number of compounds in the model building set.

$$cR^{2}p=R\times [R^{2}-{R^{2}}_{r}]$$ (6)

where cR^2^p is the coefficient of determination for Y-scrambling, R is the coefficient of determination for Y-randomization and R_r_ is the mean value of ’R’ derived from random models.

#### 2.3.1 Applicability domain (AD)

The domain of applicability was evaluated through William’s plot, which plots standardized residuals against leverage values. This aimed to ascertain whether the selected model contains compounds predominantly within the designed domain or includes outliers and influential [23, 48]. The assessment involved examining the leverage approach and the warning leverage using Eqs (7) and (8):

$$h_{i}=x_{i}{\left(X^{T}X\right)}^{-1}x_{i}^{T}$$ (7)

$$h*=\frac{3(Q+1)}{N}$$ (8)

where, h_*i*_ represents the leverage approach, X is the n×k descriptor matrix pertaining to the training sets. X^T^ is the transposed matrix employed during model creation. h* is the warning leverage. Q is the no. of descriptors in the chosen model, and N is the total number of compounds within the training sets.

### 2.4 Ligand-based drug design

The criteria guiding the selection of a lead compound for subsequent analogous design were exclusively centered around the information gained from the selected QSAR model [49]. This involved identifying the compound with the highest pIC_50_, a minimal residual value, found within the preferred applicability domain (AD), and complied with Lipinski’s rule of five, along with a favorable pharmacokinetic profile. In line with these considerations, compound **40** from the dataset emerged as the chosen lead compound. It was then subjected to modifications through the addition and replacement of various groups at specific positions (template compound). These modifications were driven by the mean effect values of the molecular descriptors used to generate the selected model [50].

### 2.5 Molecular docking studies

In order to identify the active amino acid residues and to assess type of interactions between the compounds and the SmTGR protein, molecular docking studies was performed [51, 52]. The optimized 3D-structures of all ligands were saved in the Protein Data Bank (PDB) format. The 3D structure of the SmTGR receptor (PDB ID: 6ZST), co-crystallized with a 3-(3-methoxyquinoxalin-2-yl)propanoic acid ligand with excellent resolution of 1.7 Å, was acquired from the Protein Data Bank (https://www.rcsb.org) and processed using Biovia Discovery Studio (BDS) Visualizer Version 3.5 software. This involved the removal of excess water molecules and the co-crystallized ligands from the X-ray structure prior to initiating the docking procedure [53]. Employing the Molegro Virtual Docker (MVD) 6.0 software, the lead compound, along with the newly designed compounds and PZQ, were subjected to docking within the active site of the SmTGR receptor. The docking simulation was performed with a minimum of 50 iterations to produce five potential poses. The optimal poses were then chosen using predefined scoring functions (MolDock score and Hydrogen bond energies). To examine the different intermolecular interactions in the docked complexes, BDS Visualizer was utilized [46].

### 2.6 Molecular dynamics simulations

Molecular dynamics (MD) simulation serves as a scientific approach for probing the intricate motions of molecules and atoms within dynamic systems, particularly protein-ligand complexes, with the overarching goal of gaining insights into significant physicochemical phenomena [54–56]. The 2 best designed compounds underwent a series of MD simulations lasting 100 nanoseconds. The CHARMM-GUI web-based graphical interface was employed to establish the simulation system, generating the force field for both ligands and proteins [57, 58].

The simulations, lasting 100 ns in a periodic water box, utilized the CHARMM36 force field and the Gromacs version 2020 software package [59, 60]. The complexes were placed within a rectangular box with a buffer distance of 10 in each direction [61]. Subsequently, the box was solvated by adding TIP3P water molecules. To neutralize the system’s charge for the 40D and 40J ligands, 4 Na+ ions and 0 Cl- ions were added. Additionally, 0.00 M NaCl was introduced to mimic a cellular environment. Minimization of the docked complexes was performed using the CHARMM36 force field.

Each system underwent thermal equilibration at a temperature of 310 Kelvin, involving 5000 iterations (equivalent to 10 picoseconds). The production run of the NPT ensemble extended for 100 seconds. The Lincs approach confined hydrogen, resulting in a time step of 2 fs. A switching technique with a range of 12–14 was employed to investigate van der Waals forces, with a cutoff value of 14. Long-range electrostatic interactions were calculated using the particle mesh Ewald (PME) technique, employing a maximum grid spacing of 1.2. PME calculations were performed at each iteration without a multiple-time stepping approach [62].

Temperature was maintained at a constant 310 K, and the barostat’s system size changes were set to a target of 1 bar. Numerical integration used a time interval of 2 femtoseconds. Subsequently, simulation output was adjusted, and trajectories were evaluated using VMD software, Bio3D, and QTGRACE. System stability was examined through various parameters, including root mean square deviation (RMSD), root mean square fluctuation (RMSF), radius of gyration (Rg), number of hydrogen bonds (H-bonds), principal component analysis (PCA), and dynamics cross-correlation map (DCCM) [63].

#### 2.6.1 Binding free energy calculation using MM-PBSA

In the MD simulation, free energy calculation takes a major role in determining the binding stability of ligands-protein complex [64]. In this study, the MM-PBSA method was used to calculate the free binding energy between ligands and the SmTGR enzyme. This method considers both bonded and non-bonded interactions, encompassing van der Waals and electrostatic forces. Binding free energy (ΔG) estimation was done by Eq (9) using the script MMPBSA.py of the AMBER package [57].

$$\left[\Delta G_{bind}\;G=‐complex\;G‐protein\;G–ligand\right]$$ (9)

Where, G-complex is the free energy of the complex; G-receptor is the free energy of the receptor; G-ligand is the free energy of the ligand [65].

### 2.7 Pharcokinetics and drug-likeness predictions

Following the successful docking with the SmTGR receptor, the newly developed compounds were evaluated for their potential as drug candidates, by assessing their pharmacokinetic and drug-like properties. This evaluation was carried out by utilizing the pkCSM (https://biosig.lab.uq.edu.au/pkcsm/) and Swiss-ADME (http://www.swissadme.ch/) online tools, which facilitated the assessment of their absorption, distribution, metabolism, excretion and toxicity (ADMET) profiles and drug-likeness properties [66].

## 3. Results and discussion

### 3.1 QSAR analysis

Four distinctive QSAR models were generated utilizing the GFA technique, all passing internal validation (shown below) as proposed by Umar Abdullahi Bello and co-workers [43]. Numerous researchers have employed the GFA approach in model building due to its flexibility and non-linear modeling capacity [67–69]. Aligning with benchmarks values in **Table 1**, only two of the created models satisfied the requirements for external validation against the test set compounds [70]. Among the models generated, model 2 emerged as the most suitable for predicting the inhibitory activities of the compounds and was chosen for further studies.

**Model 1** pIC_50_ *= - 1*.346 * **VE1_Dzs***—0*.425 * **nBondsM** *+ 10*.846 * **SpMax2_Bhv** *+ 2*.194 * **MLFER_E***—28*.699 * **WTPT-2** *+ 25*.*580*

**Model 2** pIC_50_ *= - 0*.444 * **nBondsM** *+ 8*.609 * **SpMax2_Bhv** *+ 2*.232 * **MLFER_E***—4*.777 * **VE1_D***—21*.929 * **WTPT-2** *+ 20*.*364*

**Model 3** pIC_50_ *= - 0*.428 * **nBondsM** *+ 10*.351 * **SpMax2_Bhv** *+ 2*.794 * **MLFER_E***—20*.769 * **WTPT-2***–0*.014 * **Zagreb** *+ 11*.*618*

**Model 4** pIC_50_ *= - 0*.432 * **nBondsM** *+ 10*.407 * **SpMax2_Bhv** *+ 2*.786 * **MLFER_E***—0*.044 * **MPC2***–21*.054 * **WTPT-2** *+ 11*.*981*

**Table 1:** Validation parameters for all generated models with their respective recommended threshold values.

Validation	Models parameters				Recommended threshold	Remark	Reference
	1	2	3	4			
**R** ^**2**^_**internal**_	0.806	0.798	0.797	0.794	> 0.6	Passed	[73]
**R** ^**2**^_**adj**_	0.777	0.767	0.767	0.763	> 0.6	Passed	[72]
**Q** ^**2**^ **cv**	0.702	0.681	0.629	0.623	> 0.6	Passed	[72]
**LOF**	0.891	0.930	0.932	0.946	Low value	Passed	[72]
**R** ^**2**^_**test**_	0.704	0.776	0.532	0.543	> 0.6	Models 3 & 4 failed	[72]

The reliability of the chosen QSAR model was assessed using the following evaluation parameters: an internal R^2^ (R^2^_internal_) of 0.798, an adjusted R^2^ (R^2^_adj_) of 0.767, a cross-validated Q^2^ (Q^2^cv) of 0.681, and a test set R^2^ (R^2^_test_) of 0.776 (**Table 1**). The R^2^_internal_ of 0.798 shows that the model can explain almost 80% of the total variance in biological activities, indicating how well the model fits the compounds in the training set. An R^2^_adj_ of 0.767 confirms the model’s reliability and suggests that it’s not overfitting, while Q^2^cv at 0.681 shows the model’s ability to predict compound activities within the training set. A notable R^2^_test_ value of 0.776 underlines the model’s proficiency in predicting activities for the test set compounds. These values indicate the reliability and predictive capability of the selected QSAR model in assessing compound activity. Additionally, validation metrics align with benchmark scores, meeting the criteria for an acceptable QSAR model, as suggested by Mouad Mouhsin and others [71–73].

Furthermore, **Table 2** indicates a detailed account of the molecular descriptors of the selected QSAR model. These descriptors, along with their associated categories, guide the molecular interpretation and the selection of suitable functional groups when designing new novel anti-schistosomiasis compounds with enhanced inhibitory effects targeting the SmTGR receptor [50]. Additionally, the numerical values of these descriptors are outlined in **S2 Table** of the supplementary materials.

**Table 2:** Interpretation and classes of the molecular descriptors within the selected model.

S/N	Symbol	Description	Class
1	**NBondsM**	Total number of bonds that have bond order greater than one (aromatic bonds have bond order 1.5).	2D
2	**SpMax2_Bhv**	Largest absolute eigenvalue of Burden modified matrix—n 2 / weighted by relative van der Waals volumes	2D
3	**MLFER_E**	Excessive molar refraction	2D
4	**VE1_D**	Coefficient sum of the last eigenvector from topological distance matrix	2D
5	**WTPT-2**	Molecular ID / number of atoms	2D

**Fig 1:**
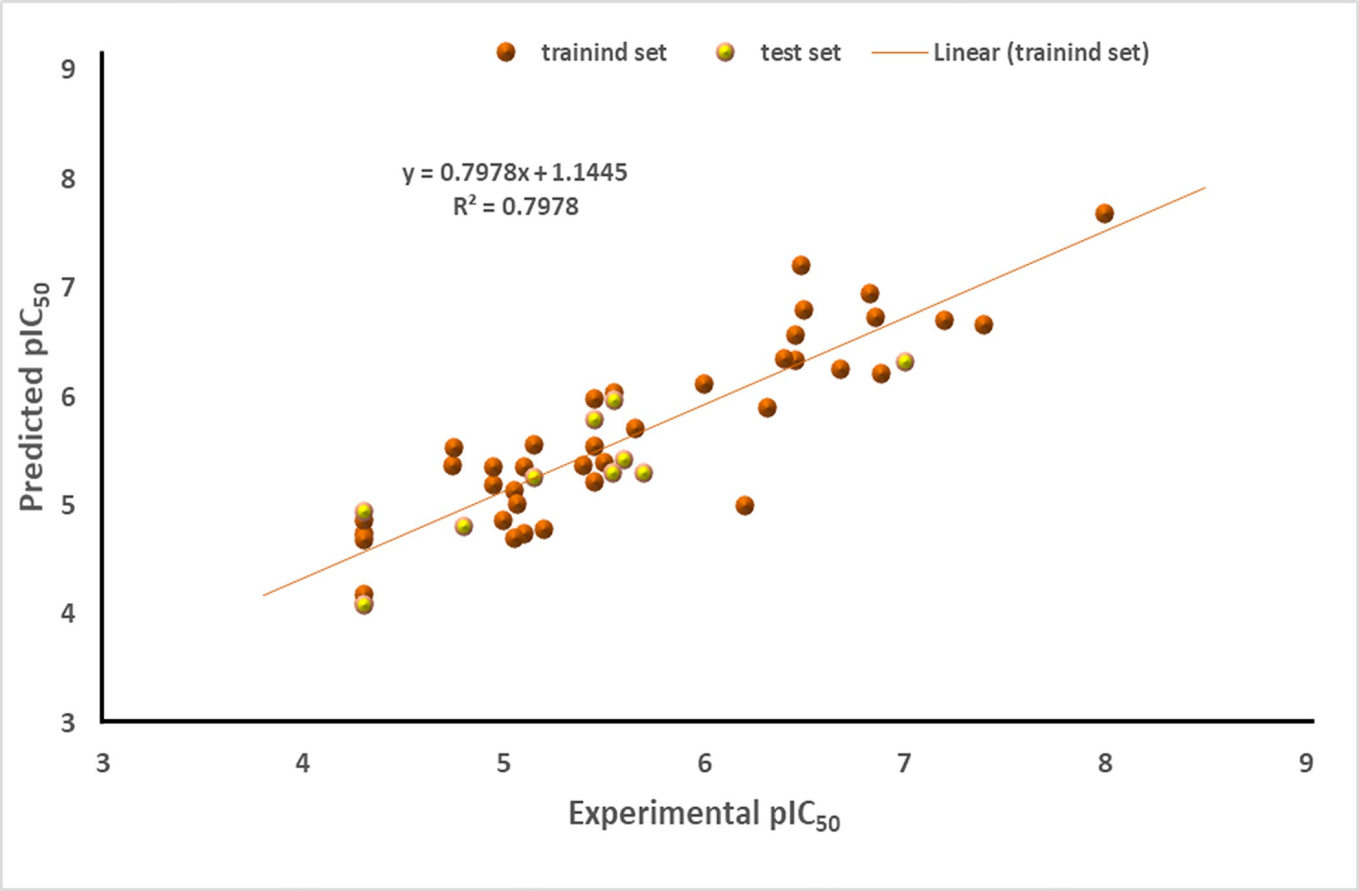
Activity plot of predicted against experimental values for SmTGR inhibition.

**Fig 2:**
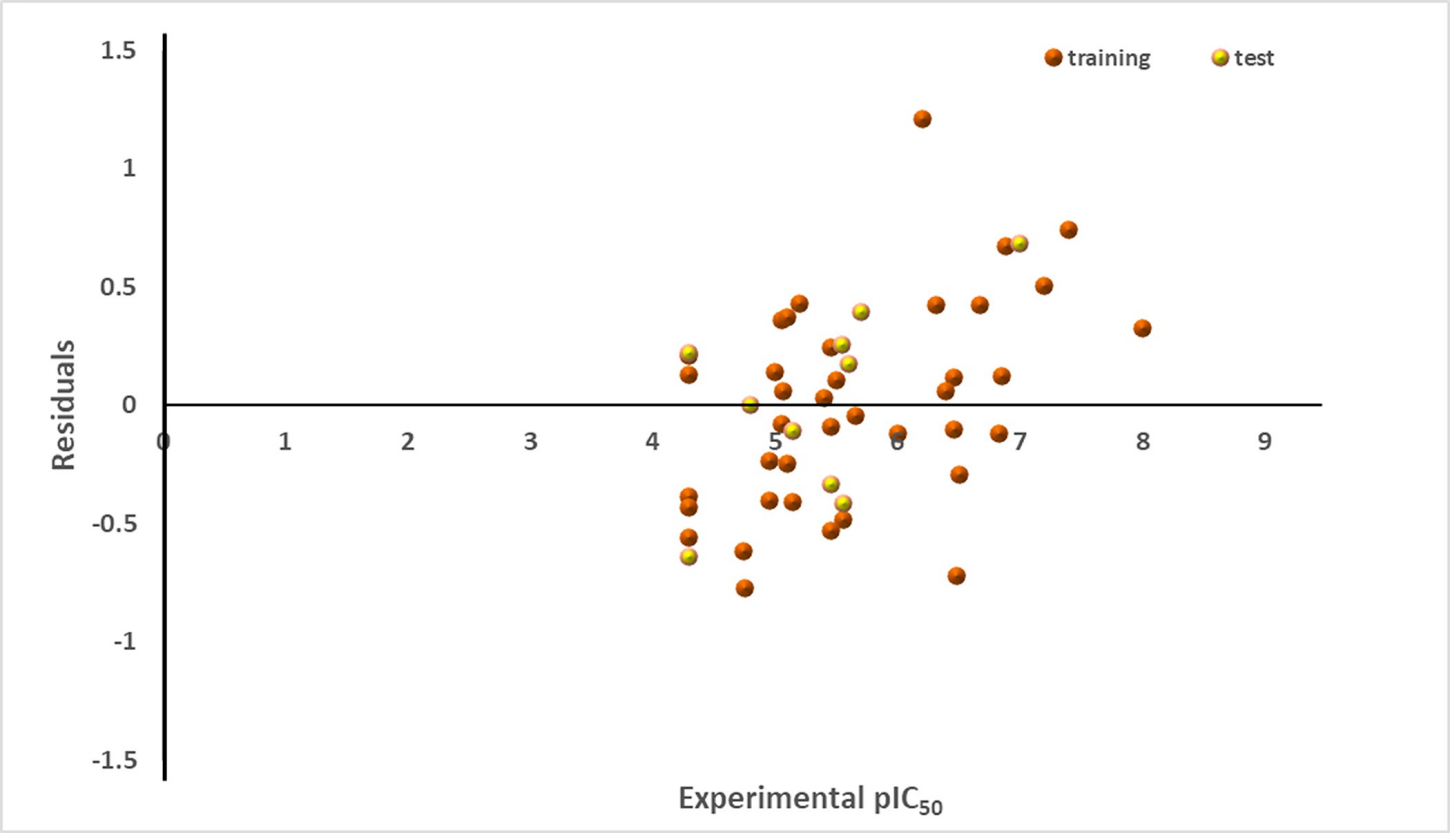
Plot of residuals against pIC_50_ values for SmTGR inhibition.

**Fig 1** illustrates an activity plot of the predicted pIC_50_ values for both the modeling and validation datasets against experimental activity values for inhibiting the SmTGR enzyme. Notably, the plot exhibits a close correspondence between these values, demonstrating minimal scattering and deviations. This alignment strongly implies the effectiveness of the model, signifying robust predictive capability. Additionally, **Fig 2** shows the residual values of the entire datasets plotted against the experimental pIC_50_ values. This graph was formulated to comprehend the disparities between the model’s estimations and the experimental data. Notably, the residuals are evenly dispersed around zero, indicating that the model’s predictions match the experimental data well. These results are consistent with the discoveries of Sagiru Abdullahi Hamza and coworkers [74].

#### 3.1.1 QSAR statistical analysis

**Fig 3:**
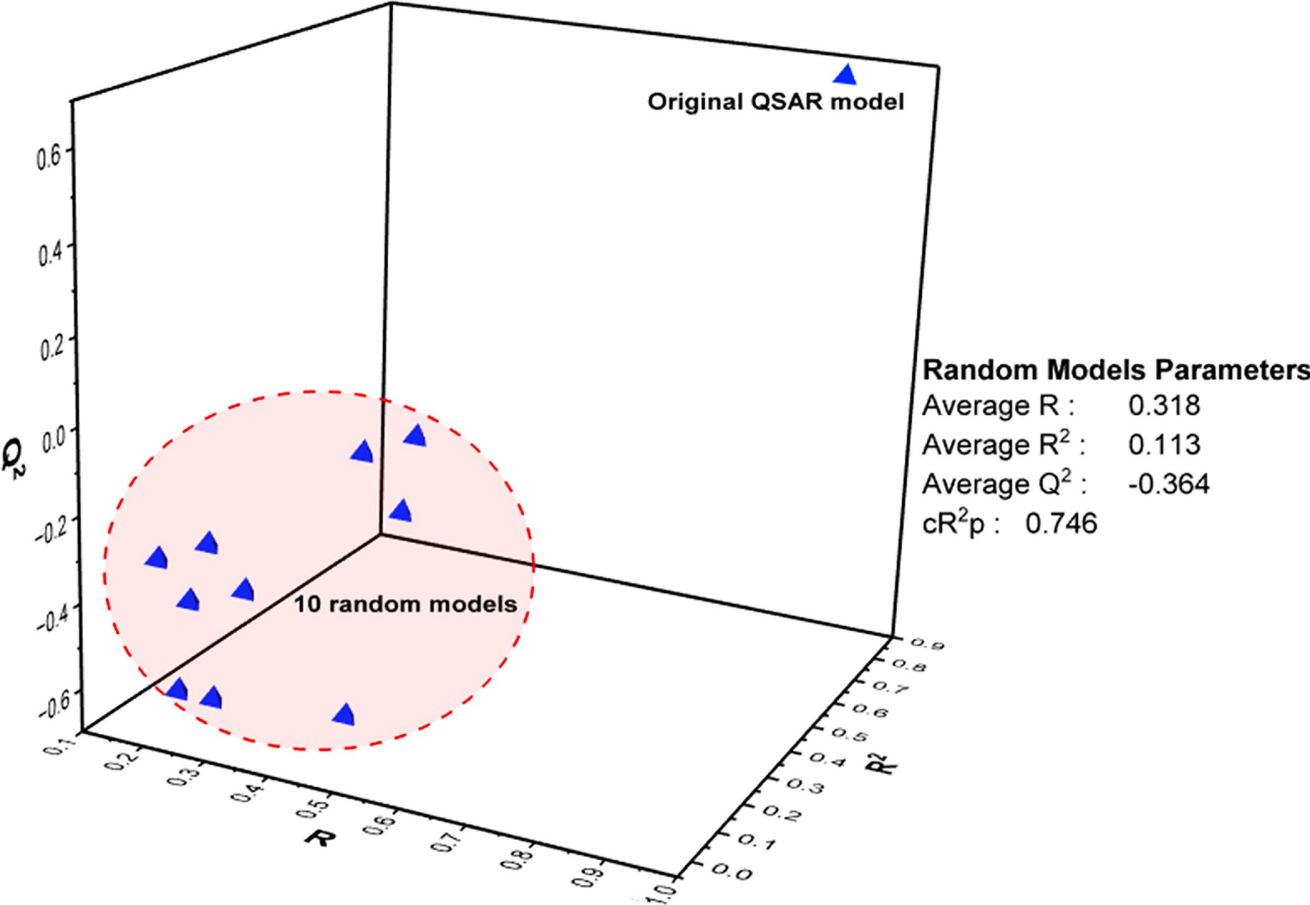
Y-scrambling assessment plot.

*3*.*1*.*1*.*1 Y-Scrambling*. To further ascertain the model’s robustness, a Y-scrambling test was conducted, involving a random reshuffling of the biological activity of training set compounds while maintaining the molecular descriptor values unchanged. As a result of this process, new models with lower performance metrics emerged. Specifically, an R^2^ value of 0.113, Q^2^ score of -0.364, and cR^2^p value of 0.746 (**Fig 3**). The lower R^2^ and Q^2^ values following the reshuffling of biological activities indicate the model’s inability to construct a suitable predictive model under such conditions [75]. Notably, the cR^2^p value of 0.746 emphasizes that the selected model isn’t merely an outcome of coincidental correlations [43]. A reliable model should exhibit notably higher R^2^ and Q^2^ values when applied to the original (unscrambled) compared to the scrambled data [76].

*3*.*1*.*1*.*2 Mean effect calculations*. From the analysis of molecular descriptors, it was observed that nBondsM, VE1_D, and WTPT-2 exhibited positive ME values, with WTPT-2 showing the highest value of 3.017. In contrast, the descriptors SpMax2_Bhv and MLFER_E displayed a negative ME values of -2.132 and -0.270, respectively (**Table 3**). Pearson’s correlation was employed to examine the interrelation between descriptors in the model. The ME values of these descriptors sum up physicochemical characteristics in a numerical format, offering distinctive structural insights for each descriptor [77]. These numeric representations serve as valuable information that can be used to enhance the compound activities. Notably, descriptors with positive coefficients namely, nBondsM, VE1_D, and WTPT-2 signify a favorable impact of these descriptors on the effectiveness of SmTGR inhibitors. This implies that higher values of these descriptors correspond to increased anti-schistosomiasis activity, and vice versa [77]. In contrast, descriptors with negative coefficients specifically, SpMax2_Bhv and MLFER_E suggest an adverse influence on the compound activities [78]. Lower values of such descriptors increases the inhibitory activities against schistosomiasis. Consequently, this underscores the significance of electron-donating groups and functional groups possessing lone electron pairs in increasing the activity of derivatives aimed at inhibiting the SmTGR enzyme.

**Table 3:** Pearson’s correlation and mean effect values of selected model.

* *	*nBondsM*	*SpMax2_Bhv*	*MLFER_E*	*VE1_D*	*WTPT-2*	*ME*
**nBondsM**	1					0.357
**SpMax2_Bhv**	0.865	1				-2.132
**MLFER_E**	0.845	0.799	1			-0.270
**VE1_D**	-0.297	-0.318	-0.143	1		0.027
**WTPT-2**	0.469	0.678	0.579	0.021	1	3.017
**∑ME**						1

**Fig 4:**
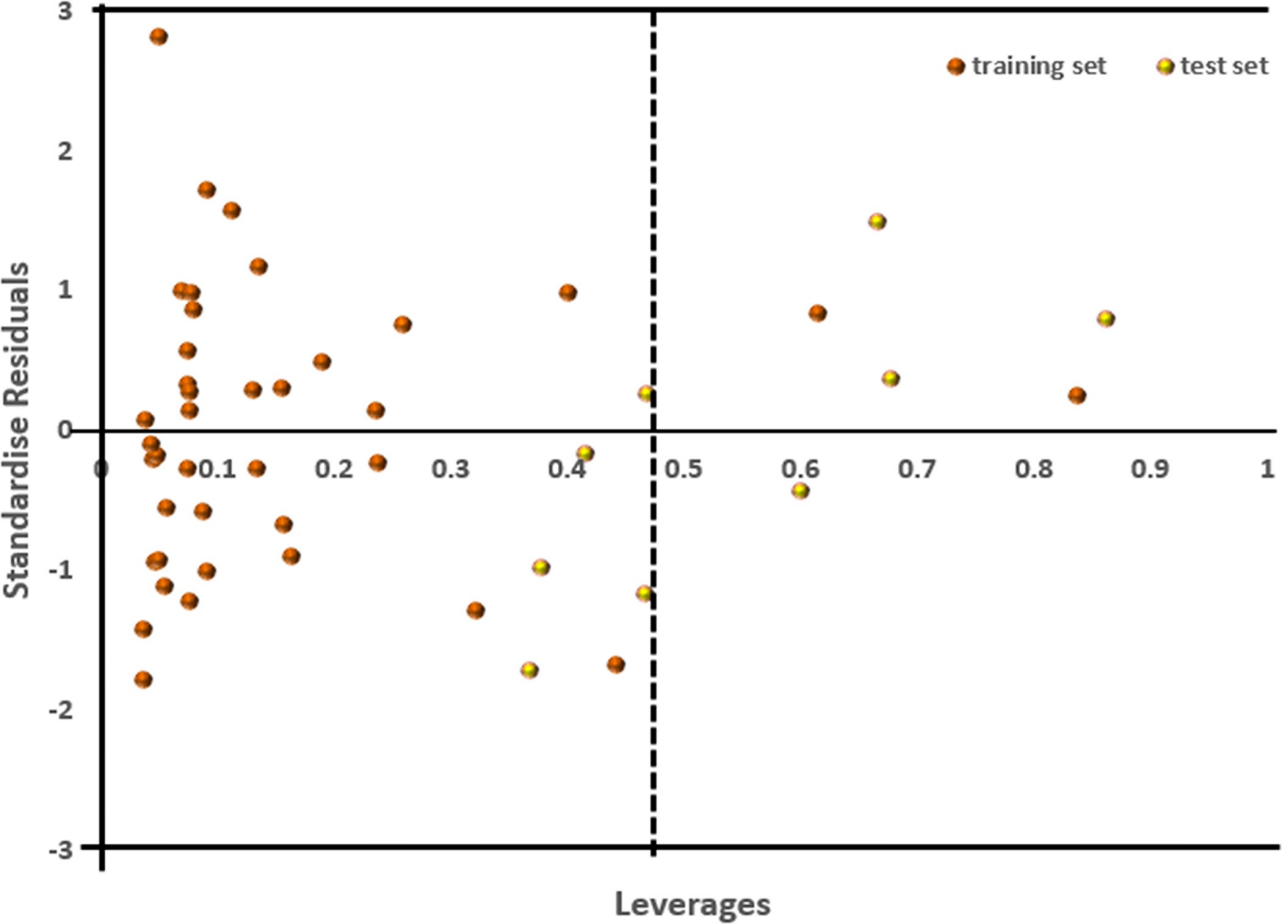
Applicability domain plot of derivatives for SmTGR inhibition.

*3*.*1*.*1*.*3 Applicability domain*. The Williams plot was utilized to identify compounds that unfavorably influence the model’s performance (**Fig 4**). This plot revealed that two compounds originating from the training set and four from the test set fell outside the preferred region, notably exceeding the warning leverage threshold (h* ˃ 0.460) (**Fig 4**) [79]. Specifically, compounds **11**, **37**, **45**, and **49** were from the test set, along with compounds **17** and **34** from the training set. These particular compounds were singled out as influential [80]. The presence of bulky substituents might account for their deviation from the desired domain. However, among the derivatives found within the predefined domain, compound **40**, having the highest pIC_50_, minimal residual value, and a favorable pharmacokinetics profile, was chosen as the lead compound.

### 3.2 Ligand-based drug design

**Fig 5:**
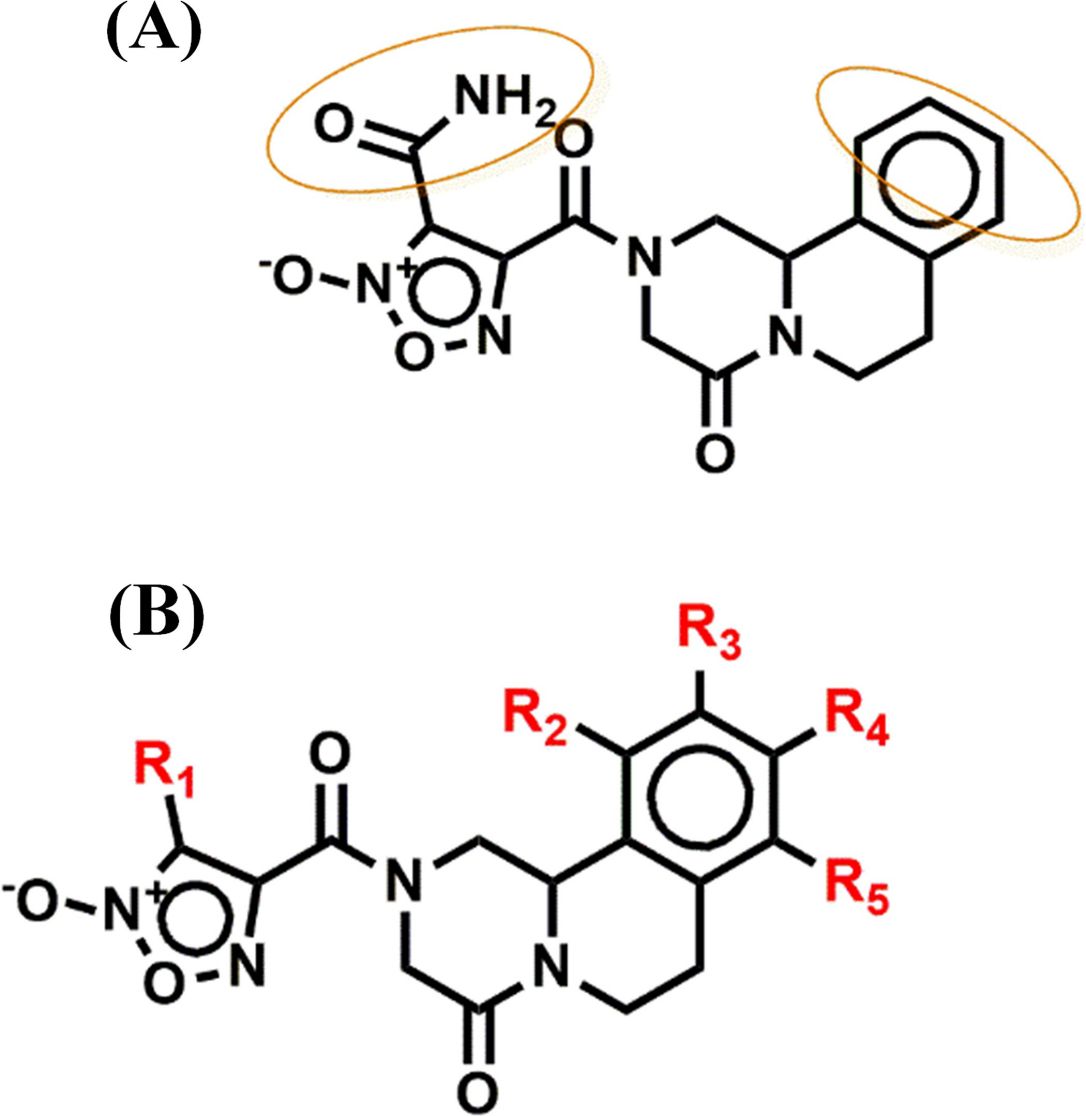
(**A)** lead compound (**40**); and (**B)** design template for novel inhibitor of SmTGR.

Compound **40** was selected as a lead compound for drug design, with various positions targeted for alterations, as indicated in the adopted template (**Fig 5**). The selection of substituents to be incorporate was guided by the SpMax2_Bhv and WTPT-2 descriptors, which were previously noted for their significant negative and positive ME values [78]. Remarkably, twelve of the newly designed analogues exhibited relatively higher activity than the lead compound. This implies that the modifications based on these descriptors led to improved compound activities, potentially making these analogues promising candidates for the treatment of schistosomiasis.

**Fig 6:**
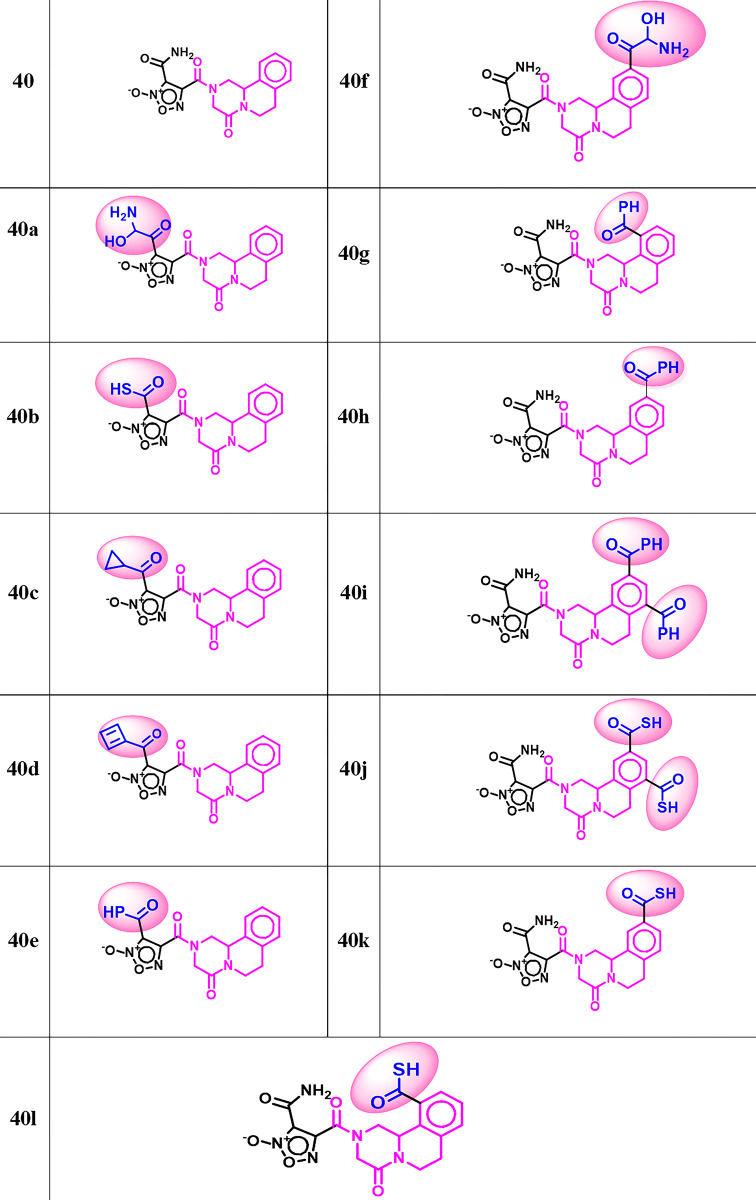
Molecular structures of newly designed potential inhibitors of SmTGR.

It was observed that introducing substituents possessing electron-donating groups (EDG) and groups containing multiple bonds holds promise for enhancing the biological activities of the derivatives [81]. The inclusion of such groups, especially those with available lone pairs of electrons, has exhibited notable increase in the efficacy of the designed compounds. Importantly, recent investigations have also validated the effectiveness of similar substituents in improving compound activities [81]. Therefore, initial structural adjustments were performed on the template structure by replacing R_1_ with various groups such as N-hydroxyamide, carbothioic S-acid, PH(CO)-, cyclopropane, and cyclobut-1,3-diene (**Table 4**) (**Fig 6**). Further modifications involving these aforementioned functional groups at positions R_2_-R_5_ have exhibited a positive impact on compounds’ activities. Notably, the introduction of these functional groups at R_1_ has elevated the predicted activities from 7.676 for the lead compound to a range of 8.100–8.331 for the newly designed compounds. Substitutions at positions R_2_-R_5_ on the aromatic ring have demonstrated a significantly stronger effect in increasing the biological activities of the potential anti-schistosomiasis agents possibly due to stronger electron density around the scaffold [82]. This effect is strikingly apparent among the newly designed entities, displaying a remarkable range of 8.537–10.076 (**Table 4**). Moreover, compound **40j**, which exhibited the highest activity, featured two carbothioic S-acid groups substituted at positions R_3_ and R_5_. This result could be attributed to an increased electron density within the ring, which in turn impacts its reactivity and electronic attributes. Collectively, all twelve of the newly designed derivatives showcased improved inhibitory effects, underscoring the potential of N-hydroxyamide, carbothioic S-acid, PH(CO)-, cyclopropane, and cyclobut-1,3-diene groups to enhance the anti-schistosomiasis properties of the derivatives.

**Table 4:** Molecular structures of newly designed potential inhibitors of SmTGR with their predicted biological activities, docking score and H-bond energies.

I.D	pIC_50_ (pred)	Moldock score/ kcal mol^-1^	H-bond energy
**40**	7.676	-150.251	-5.038
**40a**	8.331	-155.258	-7.132
**40b**	8.242	-152.256	-7.862
**40c**	8.283	-161.43	-3.007
**40d**	8.135	-170.625	-2.980
**40e**	8.100	-146.869	-11.862
**40f**	9.134	-137.018	-11.438
**40g**	8.537	-156.788	-6.613
**40h**	8.911	-158.033	-8.861
**40i**	9.652	-167.617	-7.016
**40j**	10.076	-173.613	-12.160
**40k**	9.122	-152.725	-8.488
**40l**	8.749	-147.185	-3.829
**PZQ**	6.067	-115.338	-3.314

### 3.3 Molecular docking studies

The process of docking analysis was carried out involving the lead compound, the twelve designed derivatives and the standard drug against the SmTGR receptor (PDB ID: 6ZST). Scoring functions, namely the MolDock score and hydrogen bond energies, were utilized to furnish insights into the binding energy of their interactions (**Table 4**). These scoring metrics were employed to assess the interactions between the designed analogs and the active site of the SmTGR receptor.

**Fig 7:**
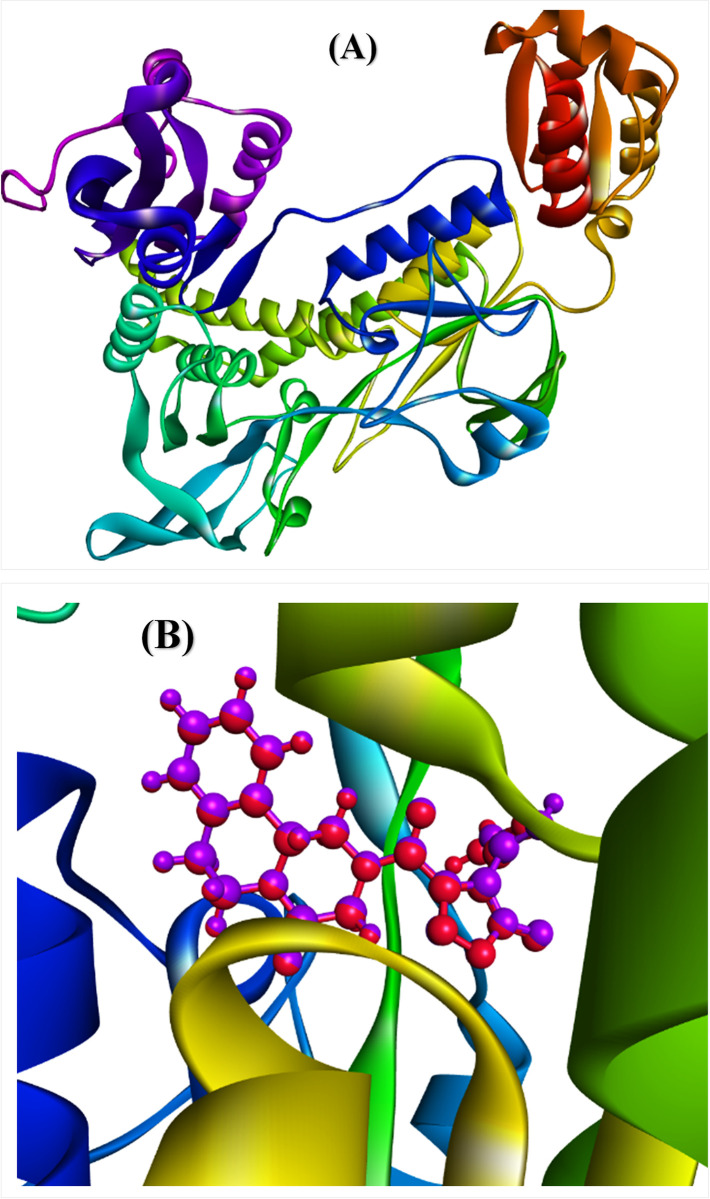
(**A**) The prepared 6ZST enzyme; (**B**) Superimposition of the lead entity.

The MVD was employed to predict the top 5 binding cavities and the most favorable binding cavity was identified by XYZ coordinates at 133.370, 9.770, and 75.030, confined within a constrained sphere of radius 20.0 Å, with a volume of 726.01 Å^3^ and a surface of 2242.560 Å^2^. The Moldock (GRID) scoring algorithm was chosen, employing a default grid resolution of 0.3 Å for 10 independent runs. Each run encompassed a maximum of 1500 iterations, utilizing a population size of 50. The default settings for pose generation and simplex evolution were consistently employed throughout the process. **Fig 7A** illustrates the alignment of the prepared 6ZST enzyme. Moreover, in order to confirm the precision of the docking algorithm and guarantee the accurate attachment of ligand molecules to the receptor’s distinct binding site, the lead compound was subjected to a second docking onto the initially docked compound. This procedure resulted in an RMSD value of 0.647 Å (**Fig 7B**). This outcome not only substantiated the reliability of the docking approach but also demonstrated its adherence to the well-established benchmark of an RMSD value of ≤2.0 Å [51].

Docking compound **40** into the optimal binding site of SmTGR revealed a MolDock score of -150.251 kcal mol^-1^ and a hydrogen bond energy of -5.038 kcal mol^-1^. The relatively strong binding energy underscores the potency of the interaction between the ligand and the receptor. Additionally, the hydrogen bond energy plays a significant role in establishing overall stability within the ligand-receptor complex. High hydrogen bond energy of -5.038 kcal mol^-1^ suggests a strong interaction between the ligand and the receptor. Abdullahi Bello Umar and co-workers have also reported that high value of Moldock score and H-bond energies increases the likelihood of the ligand being tightly bound to the receptor’s active site which can lead to a higher binding affinity, potentially resulting in a more stable and long-lasting interactions [83].

**Fig 8:**
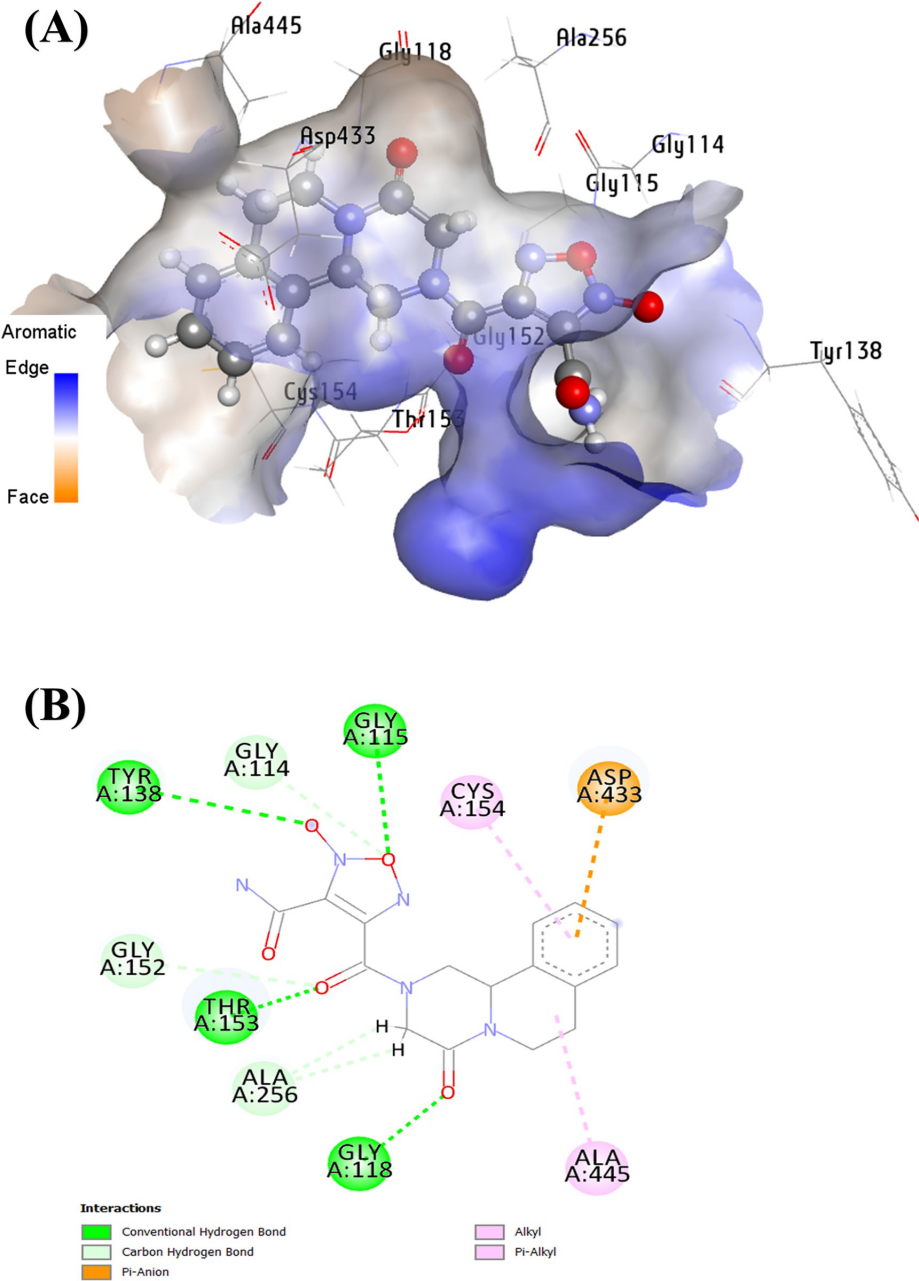
(**A**) the three-dimensional complex formed between the lead compound and 6ZST within the optimal binding cavity; (**B**) the two-dimensional interactions of the lead compound and 6ZST.

**Fig 8A and 8B** illustrated the interactions of the leading candidate (**40**) with specific amino acid residues within the SmTGR binding site: Five conventional hydrogen bonding interactions involving the oxygen atom of the oxadiazole ring, the carbonyl oxygen of the substituted piperazine scaffold, the bridged carbonyl oxygen, and the anionic oxygen of the oxadiazole scaffold with Gly115, Gly118, Thr153, and Tyr138, at distances of 2.782 Å, 2.326 Å, 1.585 Å, 1.536 Å, and 3.300 Å, respectively. Additionally, five carbon-hydrogen bonding interactions arise between the oxygen atom of the oxadiazole ring, the carbonyl oxygen of the substituted piperazine scaffold, the bridged carbonyl oxygen, and the alkyl hydrogens of the substituted piperazine ring. These interactions occur with Gly114, Gly18, Gly152, and Ala256, at distances of 2.510 Å, 3.035 Å, 2.942 Å, 2.886 Å, and 2.803 Å respectively. Furthermore, an electrostatic interaction emerges between Asp433 and the aromatic ring moiety of the ligand. Supplementary hydrophobic interactions were identified with Ala445 and Cys154 amino acid residues. Importantly, almost all the active residues of 6ZST were observed within the binding site of compound **40** with SmTGR.

**Fig 9:**
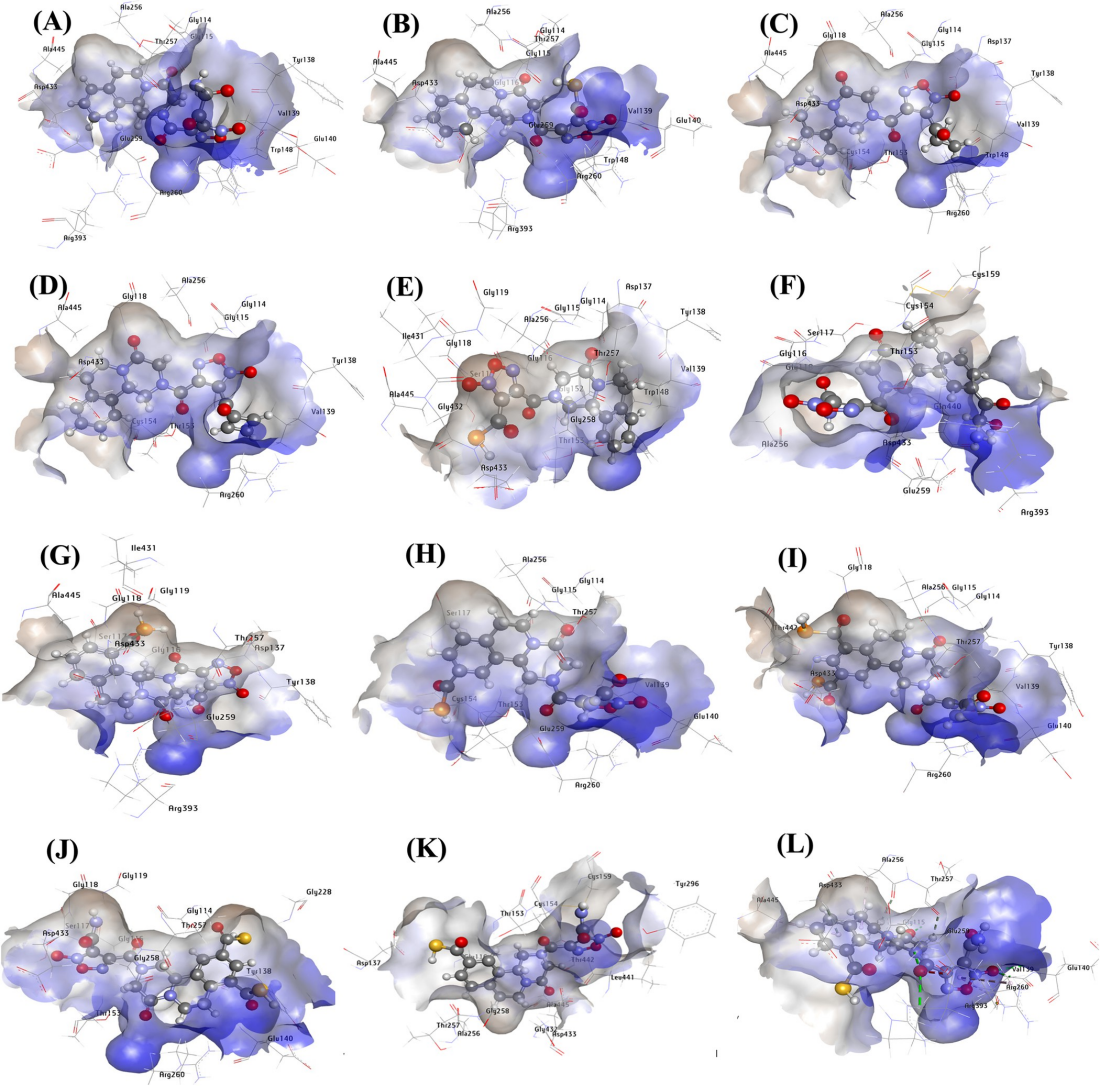
3-Dimentional interactions of designed compounds with 6ZST, (**A**) 6ZST complex with 40a; (**B)** 6ZST complex with 40b; (**C)** 6ZST complex with 40c; (**D)** 6ZST complex with 40d; (**E)** 6ZST complex with 40e; (**F)** 6ZST complex with 40f (**G)** 6ZST complex with 40g; (**H)** 6ZST complex with 40h; (**I)** 6ZST complex with 40i; (**J)** 6ZST complex with 40j; (**K)** 6ZST complex with 40k; (**L)** 6ZST complex with 40l.

Molecular docking investigations of the ligand based designed compounds yielded interesting results (**Fig 9**). The newly designed compounds with modifications at position R_1_ of the oxadiazole ring moiety (compound **40a** – **40e**) shows a significant influence on the binding affinity as they all exhibited a higher binding energy (within the range -151.869 to -170.625 kcal^-1^ and a H-bond energy range of -2.980 to -11.862 kcal mol^-1^) compared to the lead compound and PZQ with moldock score of -150.251 & 115.338 kcal mol^-1^ and H-bond energies of -5.038 kcal mol^-1^ & - 3.314 kcal mol^-1^ respectively. The incorporation of additional substituents at position R_2_ to R_5_ of the benzene ring scaffold also substantially enhanced the binding affinity of the compounds (compound **40f – 40l**) with moldock score range of -137.018 to -173.613 kcal mol^-1^ and a hydrogen bond energy range of -3.829 to -12.160 kcal mol^-1^. Interestingly, the ligand (compound **40j**) with the highest predicted activity of 10.076 was also found to have the highest binding energy and hydrogen bond of -173.613 kcal mol^-1^ and -12.160 kcal mol^-1^ respectively (**Table 4**). Compound **40j** emerged as the top-performing designed derivative due to it exhibiting the highest predicted biological activity while still maintaining remarkable stability (reflected in the moldock score and hydrogen bond energy). Compound **40j** formed numerous interactions with the active amino acid residues in the binding site of 6ZST. Specifically, it engaged in five conventional hydrogen bonding interactions between the bridged carbonyl oxygen, carbonyl oxygen of carbothioic S-acid at R^5^ position, nitrogen atom of the oxadiazole ring, Sulphur of carbothioic S-acid at R^3^ position and the carbonyl oxygen of the primary amine scaffold with Gly116, Glu140, Thr153, Gly228, Asp433 at the distance of 2.667 Å, 2.394 Å, 2.405 Å, 2.718 Å and 2.244 Å respectively. Additional six carbon hydrogen bond interactions were observed between compound **40j** and Gly114, Ser117, Gly258, Arg260, Thr257 and Ser117 at a distance of 2.170 Å, 2.035 Å, 2.829 Å, 3.055 Å, 1.382 Å and 2.282 Å. The molecular interactions of the generated PZQ derivatives are depicted in **Fig 9** and summarized in **Table 5.** The 2-dimentional interactions are presented in S1 Fig of supplementary materials.

**Table 5:** Summary of active site amino acid residues interacting with newly designed compounds.

ID	Types of interactions							
	Conventional H-bonding / Å	Carbon-hydrogen bond / Å	Electrostatic		Hydrophobic			Other
			***π-anion***	***π-cation***	***π-σ***	***Alkyl***	***π-alkyl***	
**40a**	Gly115 (2.876), Glu140 (1.926), Glu259 (3.027), Arg393 (3.092), Tyr138 (2.186)	Gly114 (3.089, 3.068), Thr257 (2.679), Ala256 (2.285)	Asp433	-	-	Ala256	Val139, Arg260, Ala445	-
**40b**	Gly115 (3.003), Gly116 (3.044), Glu140 (1.990), Glu259 (3.020), Arg393 (3.040), Thr257 (1.283)	Gly114 (3.015, 3.083), Ala256 (2.170)	Asp433	-	-	Ala256	Val139, Arg260, Ala445	-
**40c**	Gly115 (3.066), Gly118 (2.036), Thr153 (1.710), Ala256 (3.110), Asp137 (2.986), Tyr138 (3.072)	Gly114 (3.072, 2.870), Gly118 (3.007), Arg260 (2.824), Ala256 (2.757)	Asp433	-	-	Ala256, Val139	Trp148 *2, Cys154	-
**40d**	Gly115 (2.926), Gly118 (2.115), Thr153 (1.599), Tyr138 (3.300)	Gly114 (2.872), Gly118 (3.015), Ala256 (2.794)	Asp433	Arg260	-	Ala445	Cys154, Val139	-
**40e**	Gly115 (2.843), Ser117 (2.471), Gly119 (1.916), Thr153 (2.378), Asp433 (2.071), Ile431 (3.254)	Gly114 (2.039), Gly118 (2.295, 2.173), Gly152 (2.966) Thr257 (2.650, 2.687), Gly258 (2.585), Gly432 (1.963), Ala256 (2.486, 2.195), Asp137 (2.897), Tyr138 (2.805),	-	-	-	Val139	Ala256, Ala445, Val139	-
**40f**	Gly116 (2.805), Ser117 (2.568, 3.005), Gly118 (2.416), Glu259 (1.955), Arg393 (2.796), Ala256 (2.930), Gln440 (1.828, 2.310), Glu259 (2.219)	Gly116 (2.514), Arg393 (2.043), Asp433 (1.734), Gln440 (2.758)	Asp433	-	-	Cys154, Cys159	-	-
**40g**	Gly118 (1.713), Gly119 (2.507), Glu256 (2.151), Arg393 (2.814, 2.802), Thr257 (3.238), Asp137 (2.171), Ile431 (2.795)	Gly116 (2.111), Gly118 (3.081), Thr257 (2.500), Asp433 (1.200)	Asp137 Asp433	-	Ser117	Ala445	-	-
**40h**	Gly115 (2.848), Ser117 (2.595), Glu140 (1.755), Thr153 (2.310), Cys154 (2.258), Glu259 (2.760), Thr257 (2.932	Gly114 (2.127), Val139 (1.945), Thr257 (2.680, 2.141, 2.661), Ala256 (1.889)	-	Arg260	Arg260	Ala256	Val139, Arg260	-
**40i**	Gly115 (2.964), Gly118 (2.077), Glu140 (1.739), Thr442 (2.629), Tyr138 (3.286), Thr257 (2.945)	Gly114 (3.087, 2.915), Gly118 (2.717), Val139 (2.105), Arg260 (2.794), Thr257 (2.252), Ala256 (2.542), Thr257 (2.866)	Asp433	Arg260	Arg260	Val139, Arg260	-	-
**40j**	Gly116 (2.667), Glu140 (2.394), Thr153 (2.405), Gly228 (2.718), Asp433 (2.244)	Gly114 (2.170), Ser117 (2.035), Gly258 (2.829), Arg260 (3.055), Thr257 (1.382), Ser117 (2.282)	-	-	-	-	-	Tyr138 (π-lone pair)
**40k**	Thr153 (2.225), Cys154 (2.811), Tyr296 (2.093), Asp137 (2.516)	Thr153 (1.579), Gly432 (2.978), Leu441 (2.393)	Asp433	-	Gly116	Ala256, Ala445	Ala445	Cys159 (π-sulfur), Thr257 (amide-π)
**40l**	Gly115 (2.720), Glu140 (2.605), Glu259 (2.379), Arg260 (2.144), Arg393 (2.972)	Val139 (2.268), Thr257 (2.714), Ala256 (2.587, 2.018), Asp433 (3.055)	-	-	-	Ala256	Val139, Arg260, Ala445	-

### 3.4 Molecular dynamics simulations

MD simulation studies were conducted to elucidate the optimal interactions between **40d** and **40j** complexes as well as to assess their long-term stability and efficacy. The stability of the protein-ligand complexes throughout the simulation period was evaluated through the analysis of Root Mean Square Deviation (RMSD), Root Mean Square Fluctuation (RMSF), Radius of Gyration (Rg), no. of hydrogen bonds, principal component analysis (PCA), and dynamic cross correlation metric (DCCM) [84].

**Fig 10:**
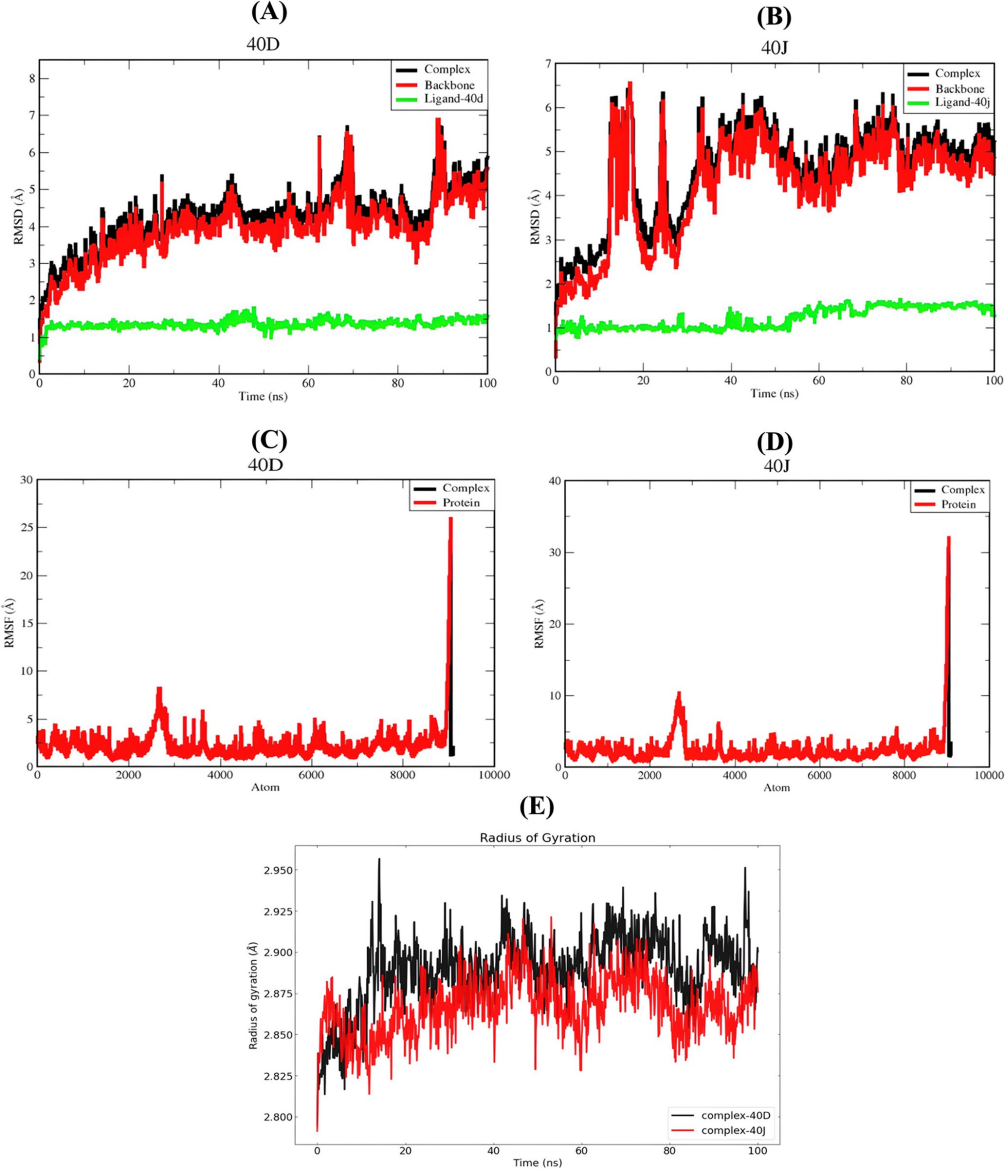
RMSD plot of (**A**) **40d**-SmTGR complex and (**B**) **40j**-SmTGR complex; RMSF plot of (**C**) **40d**-SmTGR complex and (**D**) **40j**-SmTGR complex; (**E**) Radius of gyration (Rg) plot of both complexes derived from a 100 ns MD simulation.

To gauge the stability of each protein-ligand complex, the RMSD of the protein backbone for the **40d** and **40j** complexes was calculated over a 100 ns MD trajectory, as depicted in **Fig 10**. RMSD is widely employed in structural analysis, providing insight into the stability of a given complex [85]. When analyzing a protein-ligand complex, it is crucial to consider the RMSD of the Cα atoms in the protein backbone, as it characterizes the overall conformational stability of the complex during dynamic states and simulations [86]. As illustrated in **Fig 10A**, the RMSD plot for the protein backbone’s Cα atoms exhibited an increasing pattern for the initial 30 ns, reaching a value of 4.5 Å. Notable variations were observed up to 85 ns, with pronounced fluctuations at 60 and 63 ns resulting in an elevated RMSD value of 6.5 Å. Subsequently, the RMSD showed a decreasing trend from 6.5 Å to 4 Å with slight fluctuations up to 90 ns. Although it reached the highest value around 90 ns, the RMSD decreased in the last 10 ns of the simulation, showing a stable graph line until the end. The average RMSD of the protein backbone is 3.94 Å. Contrastingly, **Fig 10B** shows an RMSD plot that increased to 3 Å for the first 10 ns, reaching its highest value of 6.5 Å after 10 ns. Notable decline occurred at 20 ns, followed by substantial increments until 45 nanoseconds. The RMSD graph then displayed ascending and descending fluctuations until the end of the simulation. Regarding the RMSD of the ligands, **Fig 10A** shows an average RMSD of 1.62 Å, while **Fig 10B** demonstrates an average RMSD of 1.19 Å. Despite the ligand in **Fig 10A** having a higher RMSD value compared to **Fig 10B**, analysis using VMD software revealed no displacement of either ligand from the protein domain. This suggests that both ligands remained stable within the binding site. To assess the stability of each protein-ligand combination, the RMSD of the protein backbone and ligand along the 100 ns MD trajectory was calculated (**Fig 10A and 10B**). The average RMSD values of the complexes are 2.12 Å and 4.6 Å, respectively. These RMSD values indicate that the protein-ligand complex in **Fig 10A** (**40d** complex) is more stable than the complex in **Fig 10B** (**40j** complex).

The average fluctuation seen in all atoms over a 100 ns MD trajectory was visualized using RMSF data for proteins and protein-ligand complexes (**Fig 10C and 10D**). PHE 181, GLY 182, TRP 183, ILE 592, VAL 593, and SER 594 showed significant alterations, possibly due to ligand binding. Both complexes (**40d** and **40j** in complex with SmTGR) exhibited similar RMSF, indicating increased stability for both complexes.

The Rg data for the protein-ligand complexes were employed to visually represent variations in structural integrity and compactness over a 100 ns MD trajectory, as illustrated in **Fig 10E**. The assessment of structural integrity and compactness relies on the Rg parameter, measuring the average distance of a group of atoms from their shared center of mass, factoring in the masses of the atoms. The **40d**-complex exhibited an average Rg value 2.80 Å greater, with the most significant deviation observed at 10 ns. Conversely, the **40j** complex had an average Rg value 2.80 Å lower, with notable deviations at 40 and 60 ns. The structural compactness and tightness of the protein complexes displayed variability during the simulation, particularly with larger fluctuations detected in the **40d** complex, suggesting both complexes maintained compact structures with **40j** being more structural integrity.

**Fig 11:**
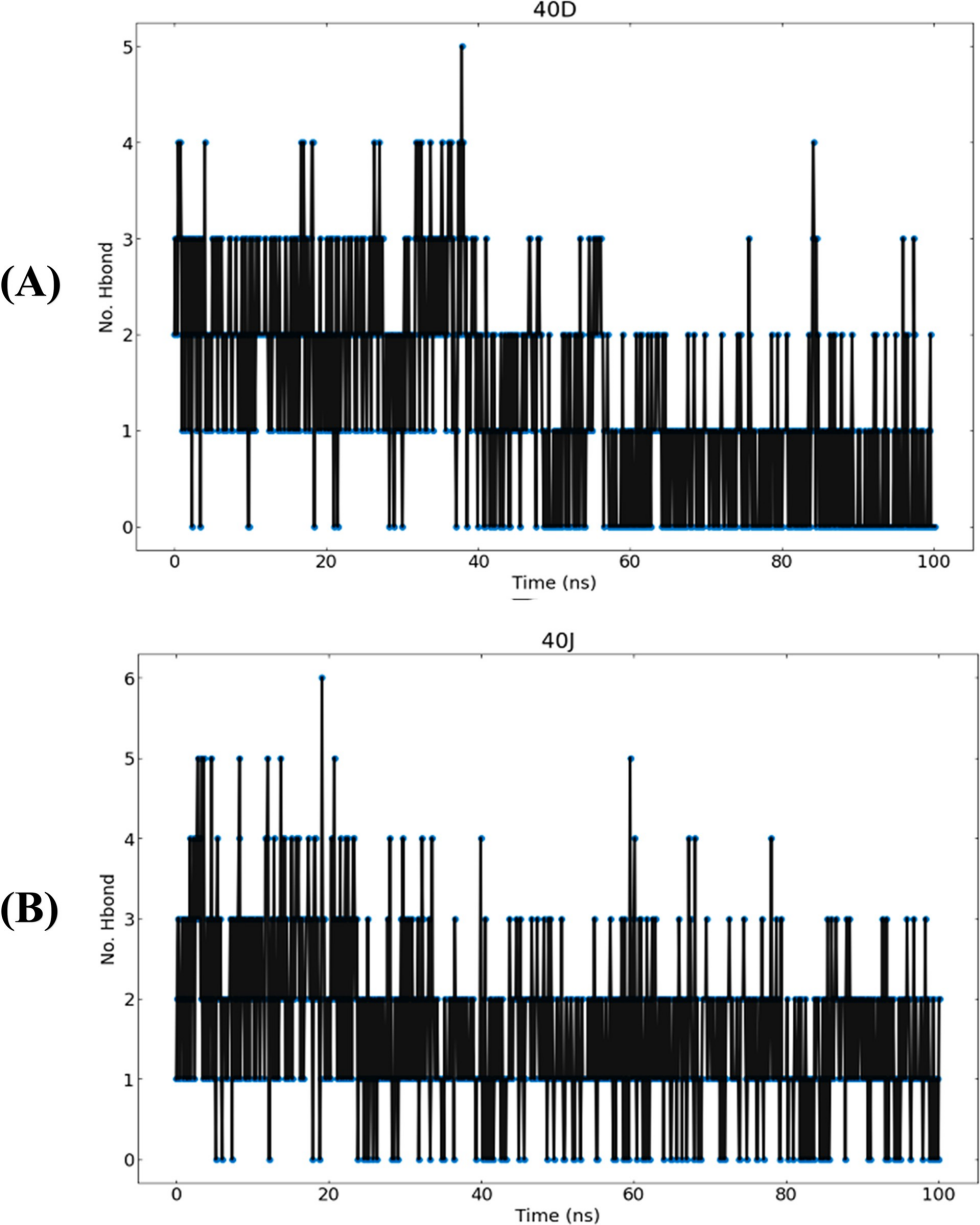
The hydrogen bond count between (**A**) **40d**-SmTGR complex and; (**B**) **40j**-SmTGR complex through the 100 ns MD simulation.

The evaluation of hydrogen bond network on the 2 complexes was conducted over a 100 ns simulation period as illustrated in **Fig 11A and 11B**. The criteria for hydrogen bonds were set as acceptor-donor distance < 0.35 nm and angle >120, with frames sampled every 2 picoseconds. The analysis revealed a greater number of stable hydrogen bonds in the docked complex, preserved throughout the MD simulations. In the case of the **40d**-SmTGR complex, stability was maintained by interactions with ASP137 and SER117 residues, with H-bond occupancies of 24.65% and 19.76%, respectively (**Table 6**). The **40j**-SmTGR complex, on the other hand, exhibited stronger hydrogen bonds, particularly involving ILE431 and TYR138 residues, with H-bond occupancies of 18.36% and 17.86% (**Table 6**). Notably, the **40d**-protein complex had a lower number of hydrogen bonds compared to **40j** complex. This shows that stronger contacts and more stable hydrogen bonding contribute to the **40j-**SmTGR complex stability during MD.

**Table 6:** Individual occupancies of detected H-bonds per ligand (40d and 40j) with SmTGR enzyme.

Donor	Acceptor	Occupancy
	**40D Complex**	
UNK1-Side-O5	ASP137-Side-OD2	24.65%
SER117-Main-N	UNK1-Side-O3	19.76%
**40J Complex**		
UNK1-Side-N1	ILE431-Main-O	18.36%
TYR138-Main-N	UNK1-Side-O6	17.86%

**Fig 12:**
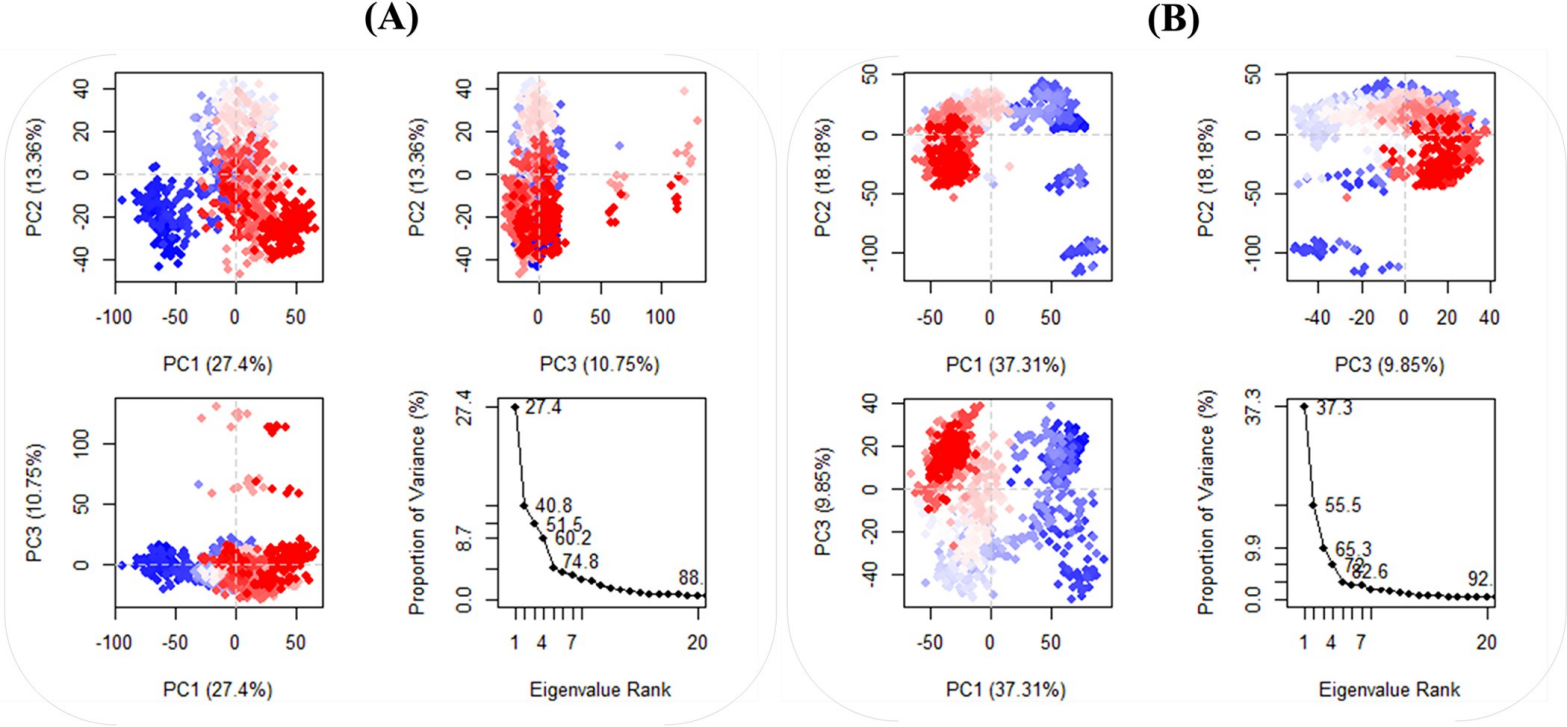
The PCA findings and eigenvalue rank plots for (**A**) **40d**-SmTGR complex and; (**B**) **40j**-SmTGR complex.

**Fig 13:**
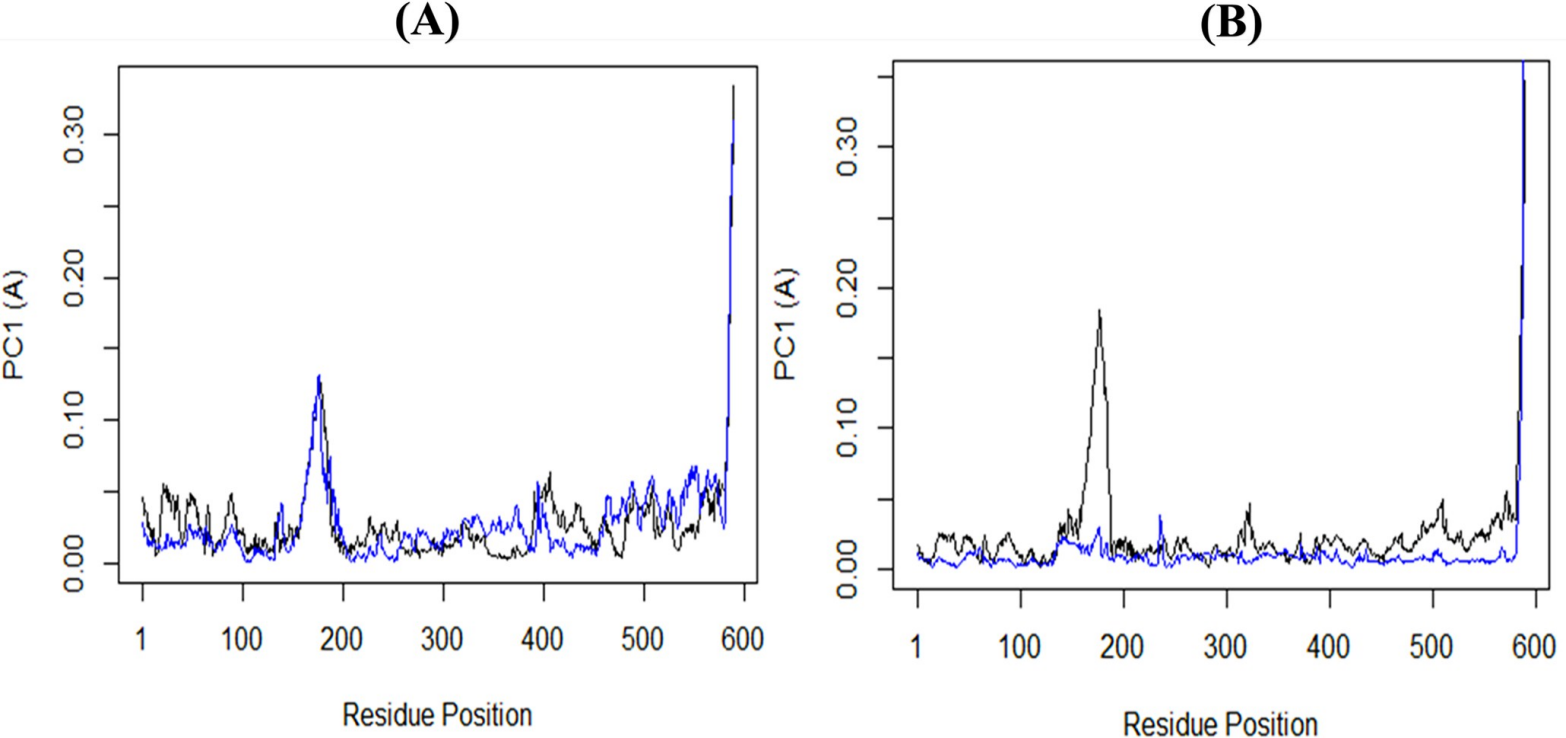
Root mean square fluctuation (RMSF) of the residual contribution to the principal component analysis (PCA). (**A**) **40d**-SmTGR complex and; (**B**) **40j**-SmTGR complex. by the black and blue lines, which correspond to PC1 & PC2 respectively.

Additionally, PCA was employed to examine the changes in the domain dynamics inside the receptor-ligand complex across a 100-nanosecond simulation period (**Fig 12**). The findings were provided on eigenfractions, representing the variance proportion obtained from a covariance matrix of 20 eigenmodels. The atomic backbone of the complex was analyzed using PCA calculations. Three conformations, namely PC1, PC2, and PC3, were used for the analysis. The normal mode molecular dynamics (MD) method was employed for these calculations. The PCA analysis demonstrated structural alterations within all clusters. The blue region displayed the most significant motions, the white region revealed moderate movements, and the red region demonstrated the least flexible movements. **Fig 12** demonstrates that the top 20 principal components (PCs) of the Ligand-**40d** and Ligand-**40j** systems accounted for 88% and 92% of the overall variation, respectively. This indicates that in comparison to the Ligand-stand system, the Ligand-**40d** system had a more limited phase space and less performance flexibility. In comparison to the PCA plots of Ligand-**40d** and Ligand-**40j**, the PC1 cluster exhibited the greatest variability, accounting for 27.4% and 37.31% of the variance, respectively. The PC2 cluster demonstrated 13.36% and 18.18% variability, while the PC3 cluster exhibited minimal variability, accounting for only 9.85% of the variance for Ligand-**40j**. In contrast to PC1 and PC2, the Ligand-**40j** cluster’s PC3 demonstrates a comparatively low degree of variability, indicating that the binding of Ligand-40j is exceptionally stable and characterized by a compact structure. Additionally, the RMSF of the residual contribution to the PCA is shown in **Fig 13** where the black and blue lines represent PC1 and PC2 respectively. The RMSF analysis revealed that PC1 and PC2 exhibited diminished flexibility when compared to the Ligand-40d.

**Fig 14:**
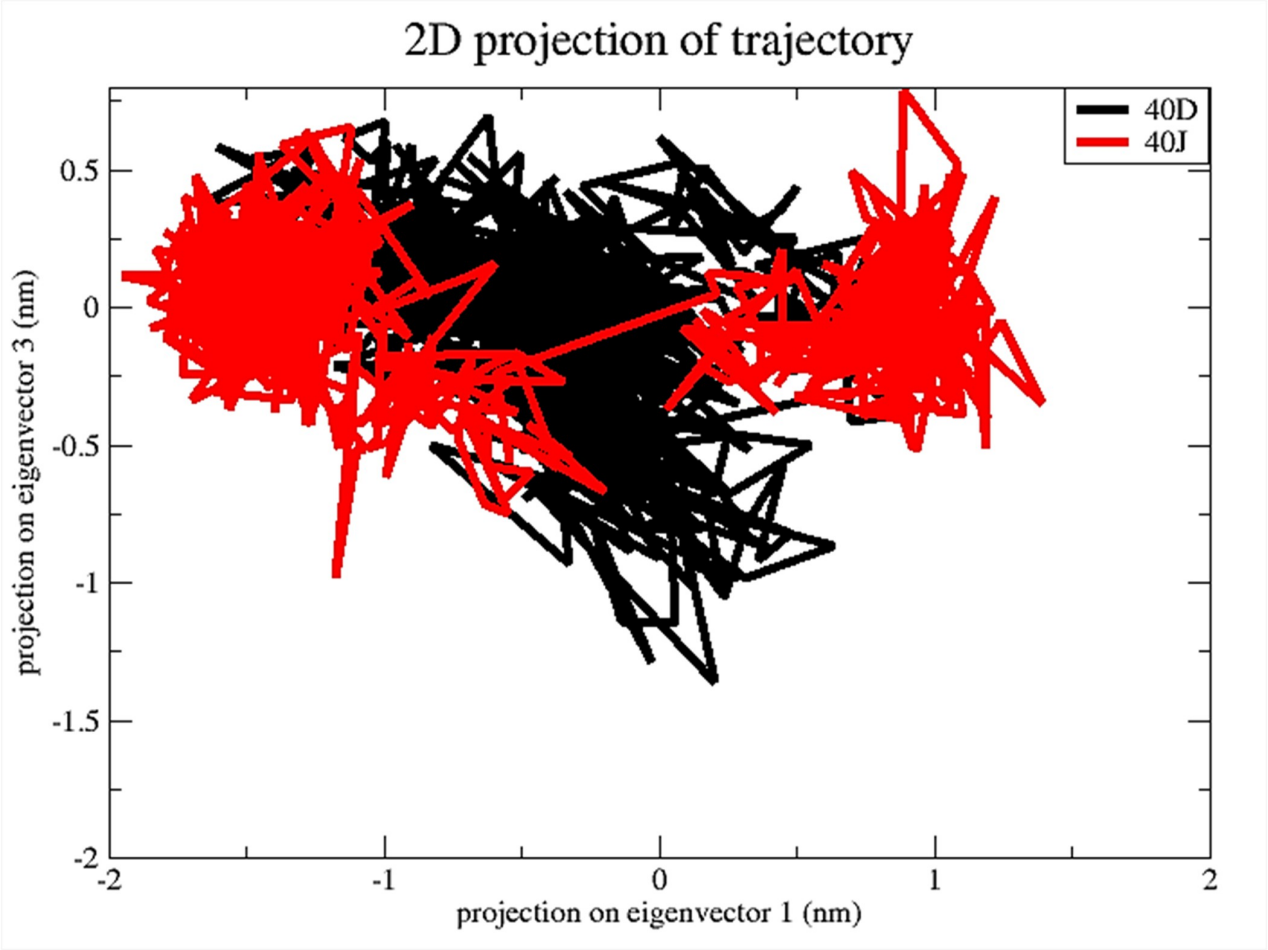
2D projections of trajectories on eigenvectors of ligands 40d, and 40j bound complexes.

In addition, we explored the kinetics of protein-ligand interactions by creating a two-dimensional projection graph using Principal Component Analysis (PCA). We analyzed the movements by utilizing the initial two principle components, PC1 and PC2. The diagram in **Fig 14** depicts the exploration of several molecular arrangements of protein-ligand complexes containing ligands 40d and **40j** within a crucial region. When looking at the 2D projection plot, the complex that fills a smaller phase space is the one that depicts the stable cluster, while the complex that occupies a larger space is the one that depicts the non-stable cluster. The outcomes of the two system simulations demonstrate that the ligand **40j** protein complex is confined to a smaller portion of phase space, while ligand **40d** occupies a considerably larger region of phase space. The results of the PCA unequivocally demonstrate that the ligand **40j** complex exhibits superior stability in comparison to the ligand **40d** complex.

**Fig 15:**
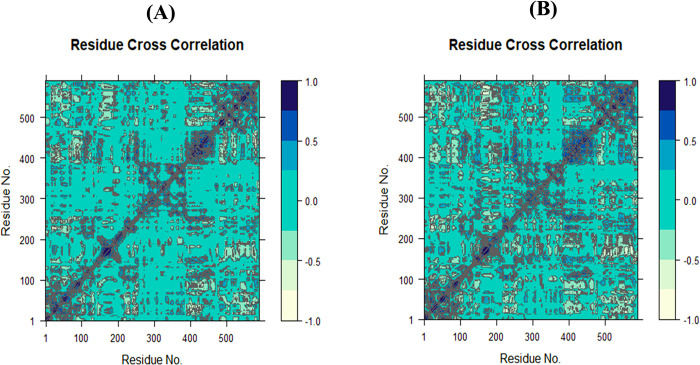
The DCCM plots for both the (**A**) **40d**-complex and (**B**) **40j**-complex.

Furthermore, the DCCM demonstrated both positive and negative impacts of amino acid correlation, displaying overall correlation in the range of −1.0 to 1.0, as shown in **Fig 15A** and **15B**. Different colors represent varying degrees of association between residues, with darker colors indicating stronger correlations. Correlations closer to 1 indicate residues moving in the same direction, while correlations closer to −1 indicate residues moving in opposite directions. Pairwise correlated graphs were constructed to examine the relationship between I and J residue indices. The analysis involved color-coding such as light green, green, and dark blue, where dark blue represents full correlation and light green represents anti-correlation. Comparing the DCCM diagrams of the two systems, it becomes evident that the correlated motions of the **40j** system were notably distinct from the **40d**-SmTGR complex. High correlation in the **40j** complex was observed compared the **40d** complex which suggests a more compact structure in the **40j** system.

#### 3.4.1 Binding free energy analysis

**Fig 16:**
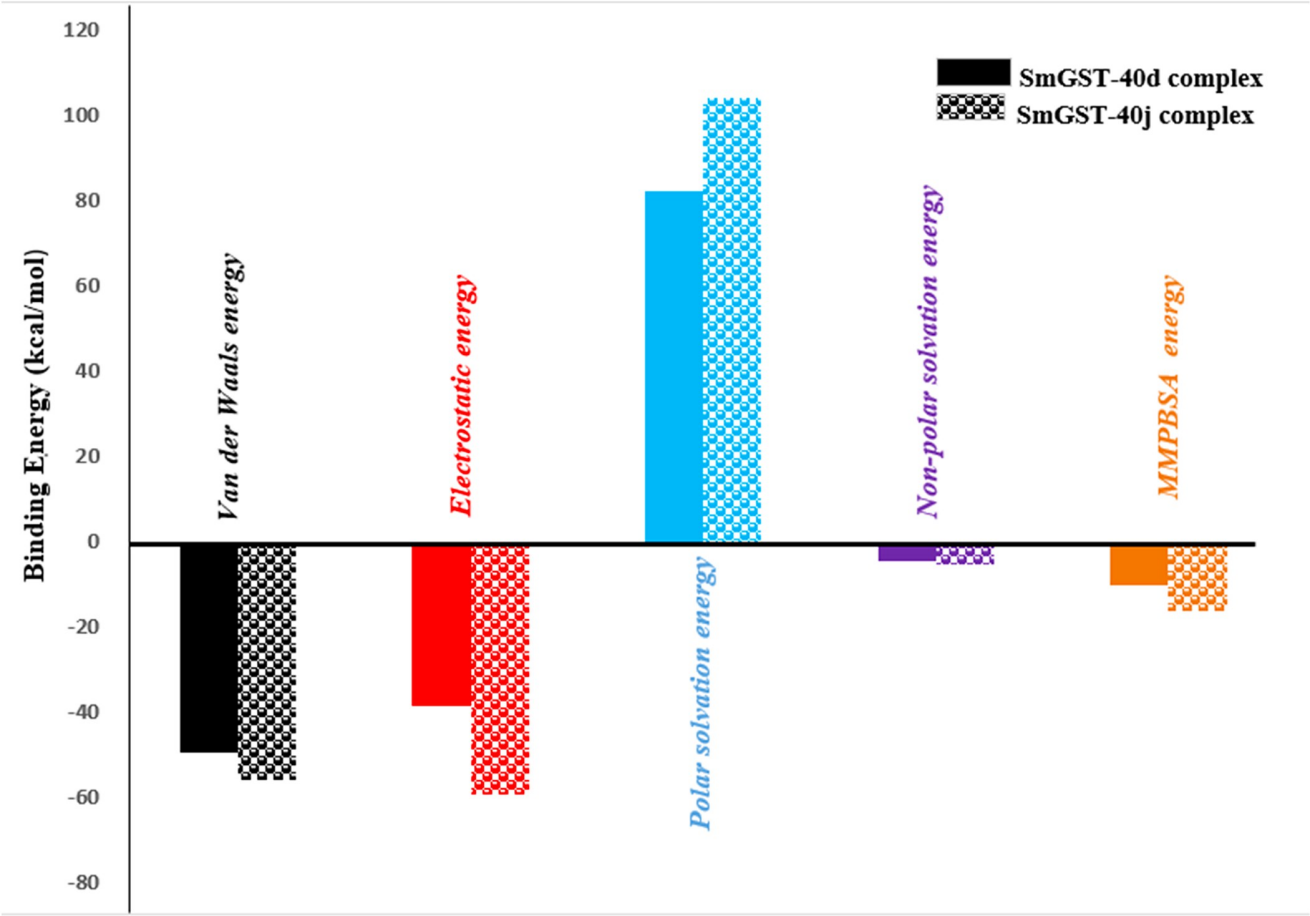
Plot of MMPBSA binding energy parameters.

The binding free energies of the protein-ligand complexes were determined based on the last 20 nanoseconds of the trajectory, employing the Molecular Mechanics Poisson-Boltzmann Surface Area (MM-PBSA) approach. A more favorable binding free energy between protein and ligands is indicated by increasingly negative values. Presented in **Fig 16** is the MMPBSA plot of **40d** and **40j** complexes. Notably, the **40j-**SmTGR complex exhibited the highest binding energy (-16.03 kcal/mol) compared to the **40d** complex (-10.19 kcal/mol). Results from the MM-PBSA study emphasized the substantial binding energy and enhanced stability of **40j** complex.

### 3.5 Drug-likeness and ADMET Analysis

The drug-likeness and pharmacokinetic ADMET properties of the designed compounds were examined to determine their suitability for therapeutic consumption [87]. A summary of the assessed drug-likeness parameters is presented in **Table 7**. Assessing these parameters is a crucial step in the early stages of drug discovery, as it links a molecule’s physical and chemical characteristics to its potential in terms of oral bioavailability and other bio-pharmaceutical properties [88]. One widely recognized preclinical guideline in drug development is Lipinski’s rule of five, which suggests that a molecule failing to meet more than two of its criteria is likely to be poorly absorbed [89]. As further discussed by Khalifah S. A and colleagues, validating the Lipinski’s rule implies that, theoretically, define the likelihood of success for a compound as a drug candidate [23]. However, it’s noteworthy that all twelve designed compounds meet these criteria, indicating their potential for pharmacological effectiveness as anti-Schistosomiasis agents.

**Table 7:** Drug-likeness assessment of designed compounds.

I.D	MW/ gmol^-1^	HBA	HBD	MlogP	Lipinski violation	Bio availability score	Synthetic accessibility	Drug likeness
40	357.32	6	1	-2.10	0	0.55	3.56	Yes
40a	387.35	8	2	-1.32	1	0.55	4.13	Yes
40b	374.37	6	0	-0.17	0	0.55	3.63	Yes
40c	382.37	6	0	0.28	0	0.55	3.89	Yes
40d	392.36	6	0	0.36	0	0.55	4.14	Yes
40e	373.28	6	0	-0.44	0	0.55	3.68	Yes
40f	430.37	9	3	-1.99	1	0.55	4.12	Yes
40g	416.30	7	1	-1.13	1	0.55	3.78	Yes
40h	416.30	7	1	-1.13	1	0.55	3.74	Yes
40i	475.29	8	1	-1.72	1	0.55	3.92	Yes
40j	477.47	8	1	-1.45	1	0.55	3.84	Yes
40k	417.40	7	1	-0.86	1	0.55	3.70	Yes
40l	417.40	7	1	-0.86	1	0.55	3.72	Yes

***Key*:** HBA: Hydrogen bond acceptor; HBD: Hydrogen bond donor; TPSA: Topological polar surface area; MlogP: Topological method

To provide a more thorough evaluation of their drug-likeness, we applied the bioavailability score (ABS) criteria. All twelve designed compounds established values of 0.55, demonstrating compliance with the rule of five and indicating an ideal bioavailability profile. Additionally, the synthetic accessibility score (SA) was evaluated, which offers insights into how easily these molecules can be synthesized. This scoring system rates the difficulty of synthesis on a scale from one (easy) to ten (very difficult). The SA scores for all proposed compounds fell within the range of 3.63 to 4.14 (**Table 7**), suggesting that these compounds are amenable to favorable and feasible synthesis [90].

ADMET predictions play a vital role in the drug development process, assisting in the selection of the most promising drug candidates, and alleviation of potential toxicity risks, ultimately enhancing the efficiency and success of drug development [91]. For the newly designed compounds, human intestinal absorption (HIA) was investigated, revealing absorption rates ranging from 43.524% to 79.637% (**Table 8**). Interestingly, all compounds displayed absorption levels exceeding the 30% threshold, indicating effective absorption in the human small intestine [91]. Moreover, the compounds were found to act as substrates for P-glycoprotein without inhibiting its function, suggesting they are likely to be transported by this efflux pump without compromising their pharmacokinetics and bioavailability [92]. Following intestinal absorption, drug molecules are distributed to their intended targets and tissues through the circulatory systems. Various parameters were predicted, including the steady-state volume of distribution (VDss), blood-brain barrier (BBB) permeability, and central nervous system (CNS) permeability. VDss values indicated that some compounds may be distributed more in plasma, while others were within a range suggesting a balance between tissue and plasma distribution [93].

**Table 8:** ADMET parameters assessment of designed compounds.

I.D	HIA	P-gp Substrate	P-gp Inhibitor	VDss	BBB	CNS	CYP3A4 Substrate	CYP3A4 Inhibitor	Total clearance	AMES toxicity	Skin sensitization
**40**	64.925	Yes	No	-0.227	-0.815	-3.210	Yes	No	0.368	No	No
**40a**	64.062	Yes	No	0.165	-1.132	-3.614	No	No	0.367	No	No
**40b**	68.454	Yes	No	-0.122	-0.914	-2.983	Yes	No	0.354	No	No
**40c**	79.637	Yes	No	0.124	-0.534	-2.794	Yes	No	0.690	No	No
**40d**	79.051	Yes	No	0.040	-0.600	-2.846	Yes	No	0.576	No	No
**40e**	73.856	Yes	No	-0.288	-0.885	-2.897	Yes	No	0.401	No	No
**40f**	43.524	Yes	No	0.158	-1.119	-4.284	No	No	0.351	No	No
**40g**	66.238	Yes	No	-0.472	-1.135	-3.383	Yes	No	0.310	No	No
**40h**	65.854	Yes	No	-0.254	-0.881	-3.351	No	No	0.327	No	No
**40i**	59.44	Yes	No	-0.686	-1.456	-3.908	Yes	No	0.369	No	No
**40j**	48.674	Yes	No	-0.186	-1.294	-4.045	Yes	No	0.596	No	No
**40k**	60.139	Yes	No	-0.202	-0.856	-3.398	Yes	No	0.438	No	No
**40l**	60.809	Yes	No	-0.246	-1.062	-3.470	Yes	No	0.425	No	No

Regarding BBB permeability, none of the compounds exhibited significant penetration (**Table 8**), suggesting limited access to the brain. However, CNS permeability predictions indicated that four compounds may moderately enter the CNS, while eight were less permeable. Importantly, for the context of targeting Thioredoxin Glutathione Reductase (TGR) from Schistosoma mansoni, crossing the BBB and affecting the CNS is generally not a critical requirement, as TGR is not associated with central nervous system functions [94].

The metabolism assessment shows predictions indicating that all but one of the twelve compounds are potential substrates for CYP3A4, which could enhance their bioavailability and reduce elimination rates [95]. However, none of the compounds were predicted to be CYP3A4 inhibitors, therefore minimizing the risk of drug-drug interactions and preserving therapeutic efficacy [96]. Total clearance significantly influences the bioavailability and half-life of drug molecules, determining appropriate dose sizes and regimens [97]. The projected total clearance of the designed compounds indicated a moderate level, with log(CLtot) ranging between 0.310 and 0.690. This suggests that the compounds could be reasonably cleared from the bloodstream by the liver [98]. Furthermore, an assessment of toxicity and skin sensitization for the proposed compounds, revealed their non-toxic nature and favorable physicochemical and pharmacokinetic ADMET properties (**Table 8**). In summary, these outcomes suggest that the proposed compounds have the potential to act as inhibitors for Schistosoma mansoni and could be considered for use in schistosomiasis treatment.

## 4. Conclusion

An *in-silico* modeling exploration was conducted on a set of 49 derivatives functioning as inhibitors against SmTGR. This study utilized QSAR, molecular docking, molecular dynamics, drug likeness and ADMET properties analyses. The reliability and predictive capability of the developed QSAR models were evaluated through statistically validation parameters. The molecular docking analysis elucidated the inhibition mechanism of the SmTGR receptor by the chosen template scaffold (compound **40**), demonstrating interactions through conventional hydrogen bonding, hydrophobic interactions, and electrostatic interactions with the active residues in the binding cavity. The findings from the QSAR modeling and docking analyses guided the design of 12 new derivatives (**40a-40l**) with improved activities and binding potentials. Molecular dynamics simulations 100 ns, affirmed the stability of the two best-designed molecules (**40d** and **40j**), within the binding cavity of the SmTGR receptor. Analysis of Root Mean Square Deviation (RMSD) and Root Mean Square Fluctuation (RMSF) plots indicated minimal fluctuations, supporting system stability as corroborated by molecular docking results. MM-PBSA calculations of binding free energy (ΔG_bind_) further validated the stability of the complexes, with **40j** emerging as the most promising among the newly designed compounds. Additionally, the designed molecules exhibited favorable results in drug-likeness and ADMET prediction analyses. The outcomes of this study suggest that these molecules could serve as promising drug candidates for schistosomiasis treatment. However, further synthesis and in vitro tests are imperative to validate the predicted properties and evaluate their potential as anti-schistosomiasis agents.

## Supporting information

S1 Table Molecular structures, experimental and predicted activities, and residual values of screened derivatives. (DOCX)

S2 Table Numerical data used to generate external R2test of chosen model 2. (DOCX)

S1 Fig 2-Dimentional interactions of designed compounds with 6ZST, (**A**) 6ZST complex with 40a; (**B)** 6ZST complex with 40b; (**C)** 6ZST complex with 40c; (**D)** 6ZST complex with 40d; (**E)** 6ZST complex with 40e; (**F)** 6ZST complex with 40f; (**G)** 6ZST complex with 40g; (**H)** 6ZST complex with 40h; (**I)** 6ZST complex with 40i; (**J)** 6ZST complex with 40j; (**K)** 6ZST complex with 40k; (**L)** 6ZST complex with 40l.
(DOCX)
