# Supplementary material for: Unveiling potent inhibitors for schistosomiasis through ligand-based drug design, molecular docking, molecular dynamics simulations and pharmacokinetics predictions
Source: PLoS One. 2024 Jun 26;19(6):e0302390. doi: 10.1371/journal.pone.0302390 (PMC11207139; doi:10.1371/journal.pone.0302390)
Supplement: S1 Table — (DOCX) [file pone.0302390.s001.docx]

**Table S1**: Molecular structures, experimental and predicted activities, and residual values of screened derivatives

| **ID** | **Molecular structure** | **Experimental pIC_50_** | **Predicted pIC_50_** | **Residual** |
| --- | --- | --- | --- | --- |
| 1 | 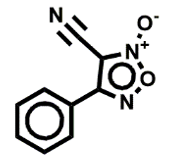 | 5.201 | 4.772 | 0.429 |
| 2 | 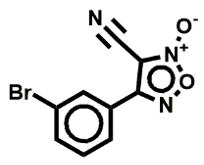 | 5.553 | 5.966 | -0.414 |
| 3 | 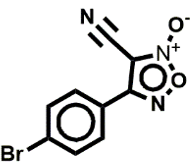 | 5.456 | 5.791 | -0.336 |
| 4 | 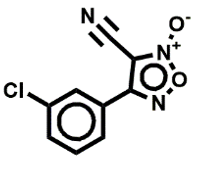 | 5.456 | 5.547 | -0.091 |
| 5 | 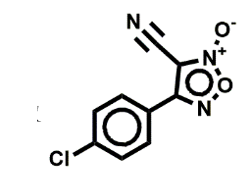 | 5.398 | 5.365 | 0.033 |
| 6 | 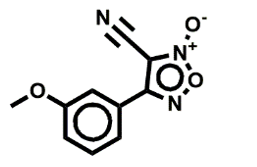 | 5.051 | 5.128 | -0.078 |
| 7 | 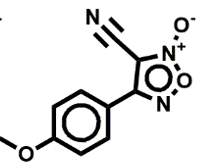 | 5 | 4.863 | 0.137 |
| 8 | 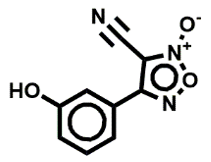 | 5.149 | 5.558 | -0.409 |
| 9 | 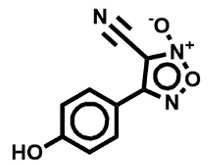 | 4.747 | 5.362 | -0.614 |
| 10 | 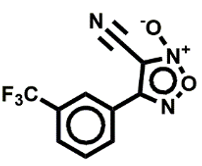 | 5.602 | 5.425 | 0.177 |
| 11 | 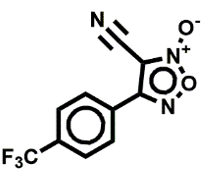 | 5.149 | 5.259 | -0.11 |
| 12 | 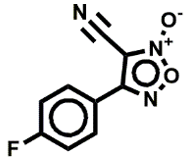 | 5.102 | 4.732 | 0.371 |
| 13 | 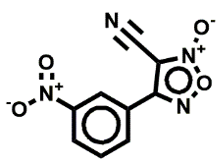 | 5.658 | 5.705 | -0.047 |
| 14 | 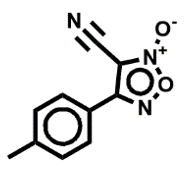 | 4.951 | 5.189 | -0.238 |
| 15 | 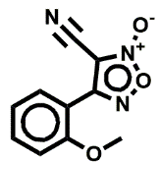 | 5.102 | 5.352 | -0.25 |
| 16 | 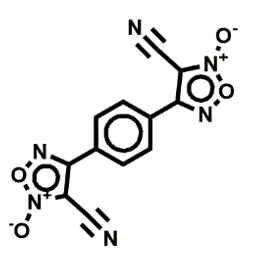 | 6 | 6.12 | -0.12 |
| 17 | 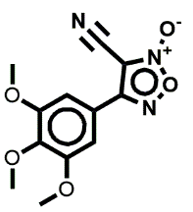 | 5.051 | 4.691 | 0.359 |
| 18 | 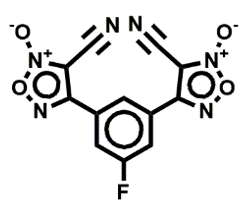 | 6.319 | 5.896 | 0.423 |
| 19 | 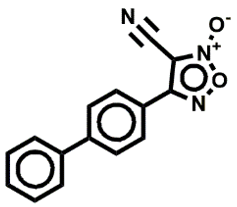 | 4.801 | 4.801 | 0 |
| 20 | 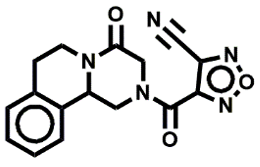 | 4.301 | 4.093 | 0.208 |
| 21 | 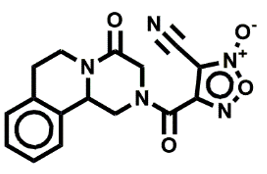 | 6.5 | 6.796 | -0.296 |
| 22 | 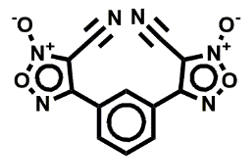 | 5.456 | 5.984 | -0.528 |
| 23 | 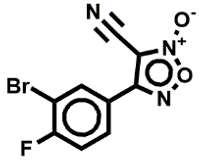 | 5.553 | 6.039 | -0.486 |
| 24 | 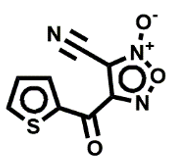 | 7.201 | 6.699 | 0.502 |
| 25 | 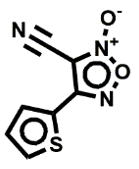 | 5.456 | 5.215 | 0.241 |
| 26 | 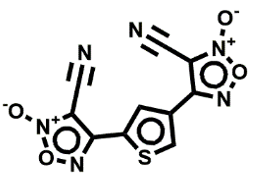 | 6.456 | 6.337 | 0.119 |
| 27 | 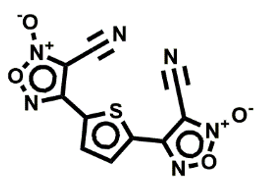 | 6.398 | 6.34 | 0.058 |
| 28 | 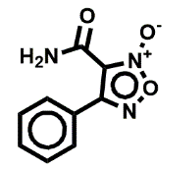 | 4.75 | 5.524 | -0.774 |
| 29 | 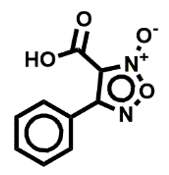 | 6.201 | 4.99 | 1.211 |
| 30 | 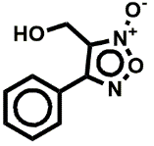 | 4.951 | 5.351 | -0.401 |
| 31 | 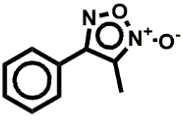 | 4.301 | 4.735 | -0.434 |
| 32 | 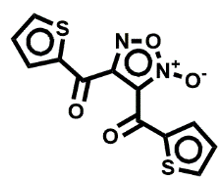 | 7.398 | 6.66 | 0.738 |
| 33 | 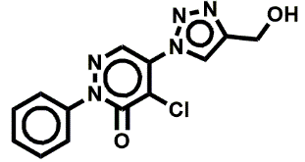 | 5.547 | 5.291 | 0.255 |
| 34 | 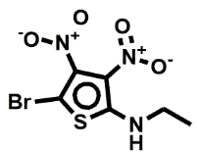 | 5.502 | 5.395 | 0.107 |
| 35 | 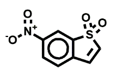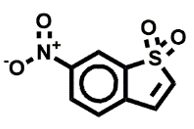 | 5.697 | 5.301 | 0.396 |
| 36 | 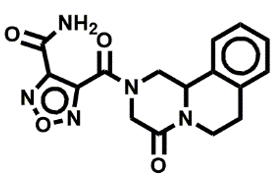 | 4.301 | 4.939 | -0.638 |
| 37 | 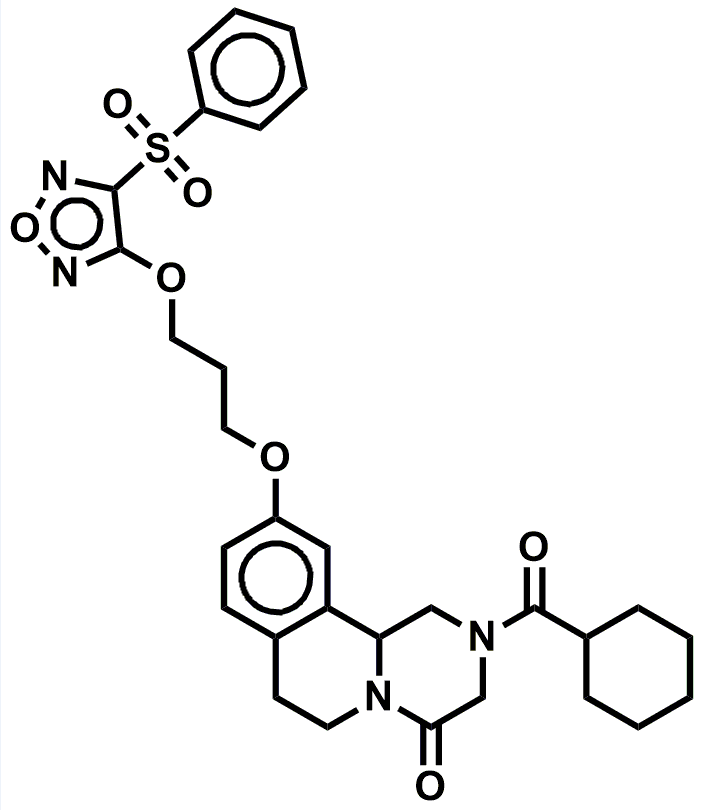 | 4.301 | 4.861 | -0.56 |
| 38 | 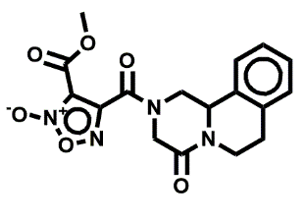 | 6.830 | 6.952 | -0.122 |
| 39 | 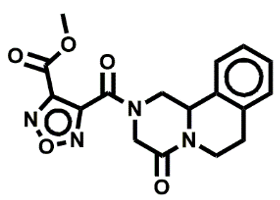 | 4.301 | 4.175 | 0.126 |
| 40 | 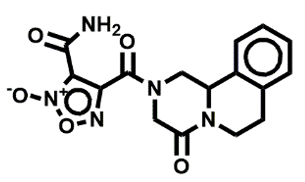 | 8 | 7.676 | 0.324 |
| 41 | 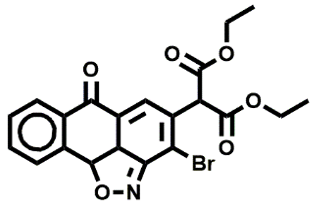 | 6.854 | 6.732 | 0.122 |
| 42 | 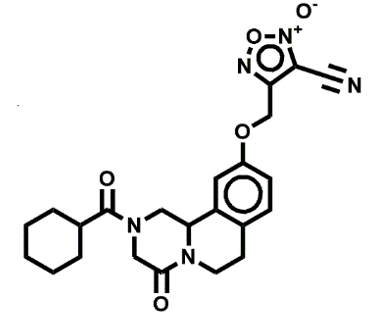 | 6.456 | 6.561 | -0.105 |
| 43 | 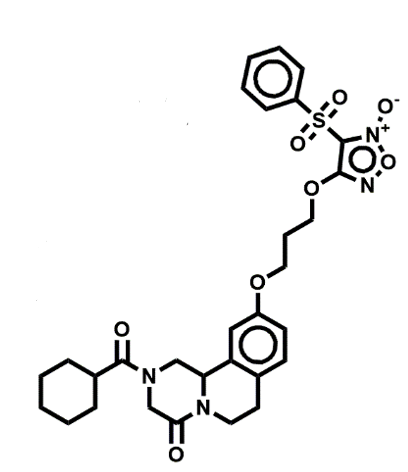 | 5.071 | 5.013 | 0.058 |
| 44 | 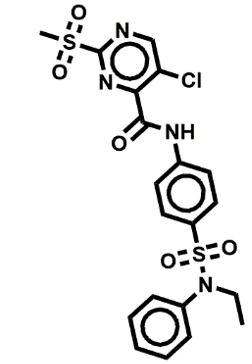 | 6.678 | 6.253 | 0.425 |
| 45 | 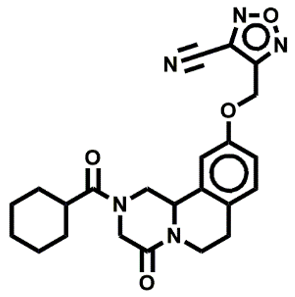 | 4.301 | 4.079 | 0.222 |
| 46 | 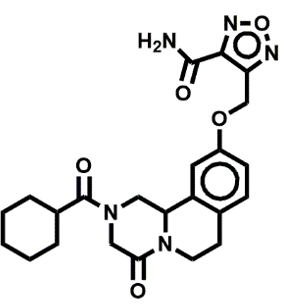 | 4.301 | 4.689 | -0.388 |
| 47 | 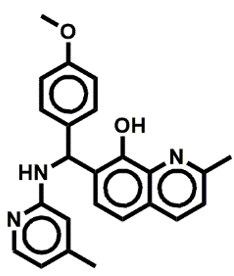 | 6.886 | 6.213 | 0.673 |
| 48 | 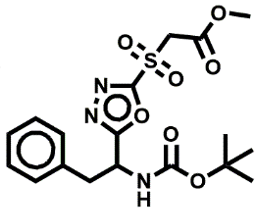 | 6.481 | 7.205 | -0.724 |
| 49 | 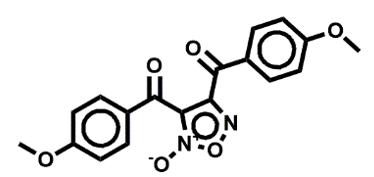 | 7.000 | 6.319 | 0.681 |
