# Supplementary material for: Unveiling potent inhibitors for schistosomiasis through ligand-based drug design, molecular docking, molecular dynamics simulations and pharmacokinetics predictions
Source: PLoS One. 2024 Jun 26;19(6):e0302390. doi: 10.1371/journal.pone.0302390 (PMC11207139; doi:10.1371/journal.pone.0302390)
Supplement: S2 Table — (DOCX) [file pone.0302390.s002.docx]

**Table S2**: Numerical data used to generate external R^2^_test_ of chosen model 2

| **ID** | **nBondsM** | **SpMax2_Bhv** | **MLFER_E** | **VE1_D** | **WTPT-2** | **Y_exp_** | **Y_pred_** | **Y_exp_-Y_pred_** | **Y_exp_-Y_m_** | **(Y_exp_-Y_pred_)^2^** | **(Y_exp_-Y_m_)^2^** |
| --- | --- | --- | --- | --- | --- | --- | --- | --- | --- | --- | --- |
| 2 | 10 | 3.57 | 1.610 | 0.055 | 2.008 | 5.553 | 5.966 | -0.414 | -0.108 | 0.171 | 0.012 |
| 3 | 10 | 3.574 | 1.610 | 0.099 | 2.008 | 5.456 | 5.791 | -0.336 | -0.205 | 0.113 | 0.042 |
| 10 | 10 | 3.590 | 1.010 | 0.025 | 1.986 | 5.602 | 5.425 | 0.177 | -0.059 | 0.031 | 0.003 |
| 11 | 10 | 3.598 | 1.010 | 0.073 | 1.986 | 5.149 | 5.259 | -0.11 | -0.512 | 0.012 | 0.262 |
| 19 | 17 | 3.781 | 2.174 | 0.088 | 2.052 | 4.801 | 4.801 | 0 | -0.859 | 0 | 0.738 |
| 33 | 17 | 3.760 | 2.244 | 0.033 | 2.041 | 5.547 | 5.291 | 0.255 | -0.114 | 0.065 | 0.013 |
| 35 | 10 | 3.493 | 1.680 | 0.146 | 1.995 | 5.697 | 5.301 | 0.396 | 0.036 | 0.156 | 0.001 |
| 36 | 14 | 3.698 | 2.073 | 0.091 | 2.063 | 4.301 | 4.939 | -0.638 | -1.360 | 0.407 | 1.848 |
| 45 | 14 | 3.688 | 2.043 | 0.166 | 2.079 | 4.301 | 4.079 | 0.222 | -1.360 | 0.049 | 1.848 |
| 49 | 16 | 3.804 | 2.104 | 0.015 | 2.021 | 7.000 | 6.319 | 0.681 | 1.339 | 0.464 | 1.794 |
| ∑ |  |  |  |  |  |  |  |  |  | 1.469 | 6.563 |
|  |  |  |  |  |  |  |  |  |  | 0.224 |  |
| R^2^_test_ |  |  |  |  |  |  |  |  |  | 0.776 |  |
