# Supplementary material for: Unveiling potent inhibitors for schistosomiasis through ligand-based drug design, molecular docking, molecular dynamics simulations and pharmacokinetics predictions
Source: PLoS One. 2024 Jun 26;19(6):e0302390. doi: 10.1371/journal.pone.0302390 (PMC11207139; doi:10.1371/journal.pone.0302390)
Supplement: S1 Fig — 2-Dimentional interactions of designed compounds with 6ZST, (A) 6ZST complex with 40a; (B) 6ZST complex with 40b; (C) 6ZST complex with 40c; (D) 6ZST complex with 40d; (E) 6ZST complex with 40e; (F) 6ZST complex with 40f; (G) 6ZST complex with 40g; (H) 6ZST complex with 40h; (I) 6ZST complex with 40i; (J) 6ZST complex with 40j; (K) 6ZST complex with 40k; (L) 6ZST complex with 40l. (DOCX) [file pone.0302390.s003.docx]

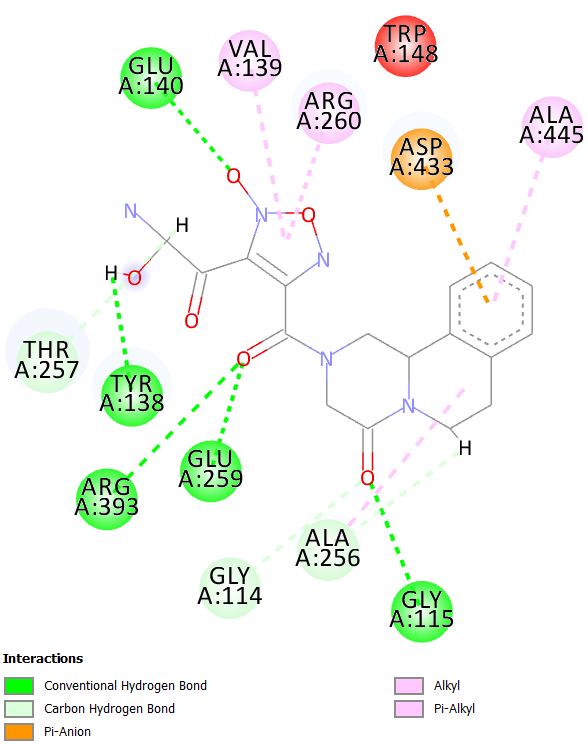


**A**


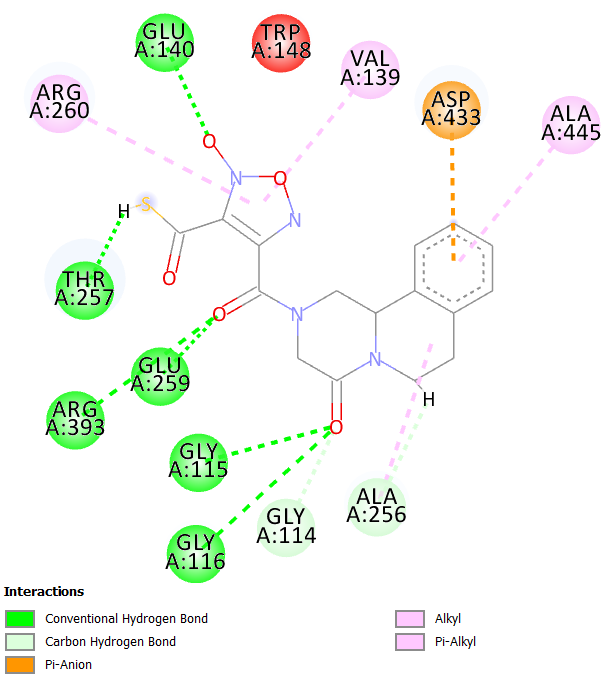


**B**


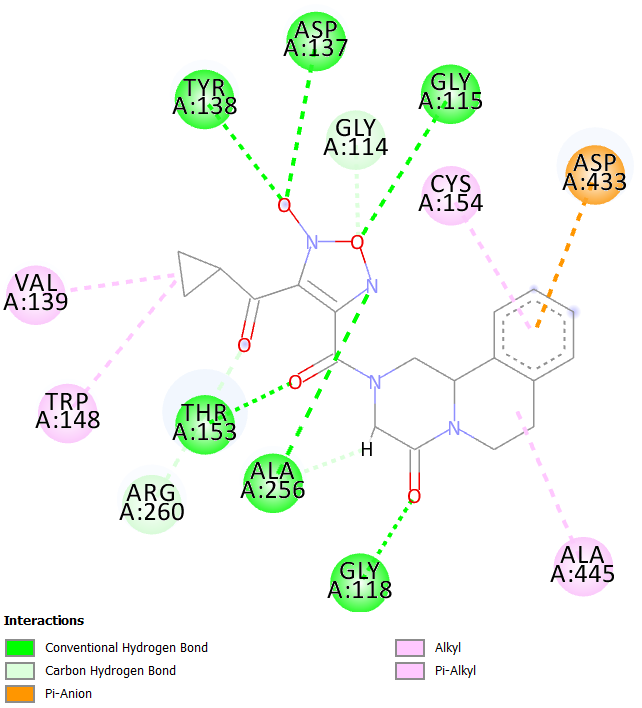


**C**


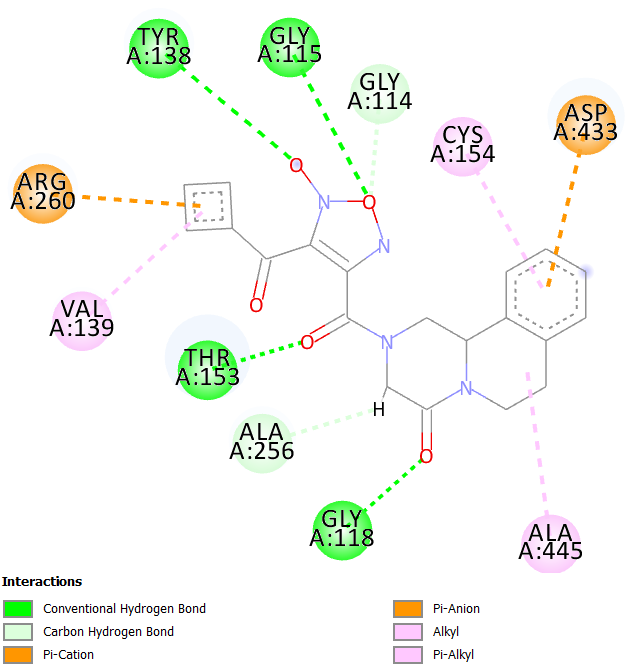


**D**


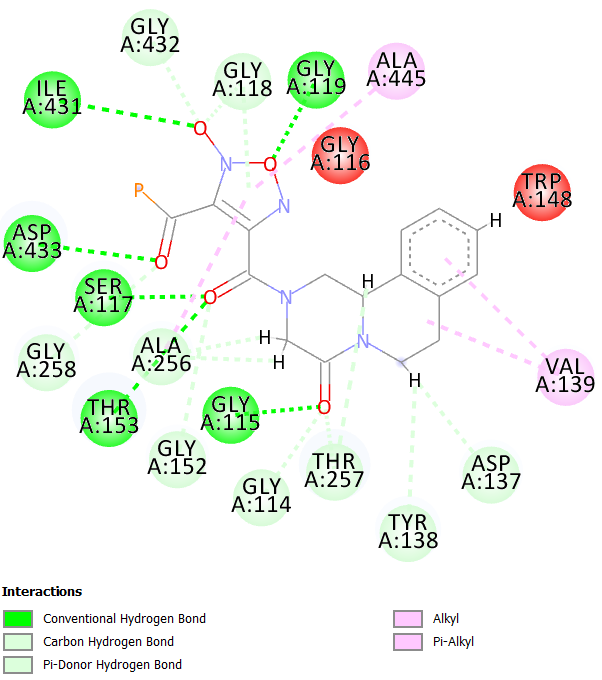


**E**


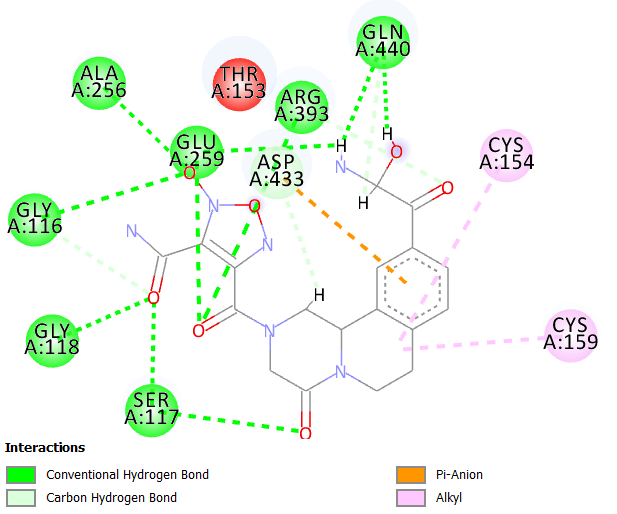


**F**


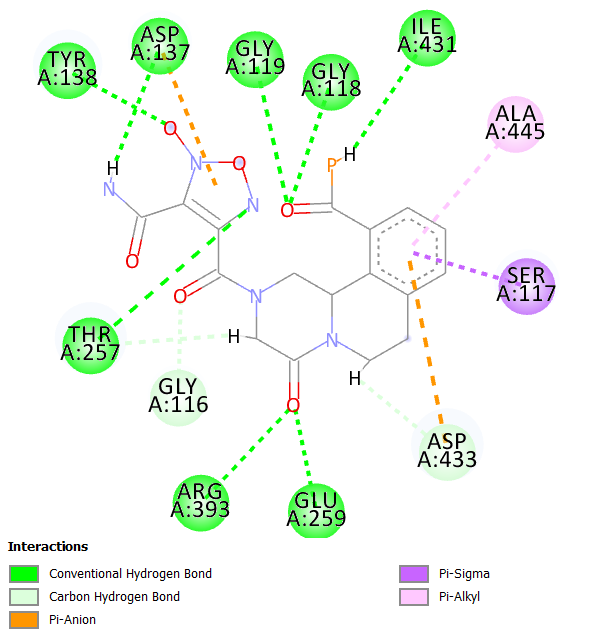

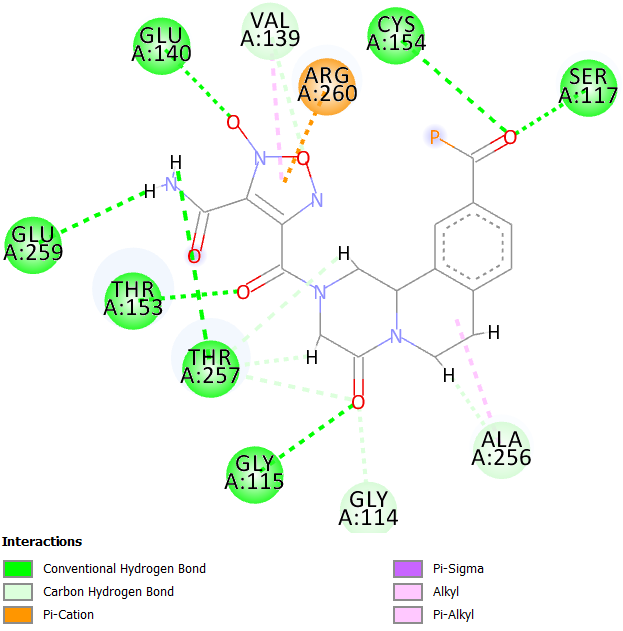

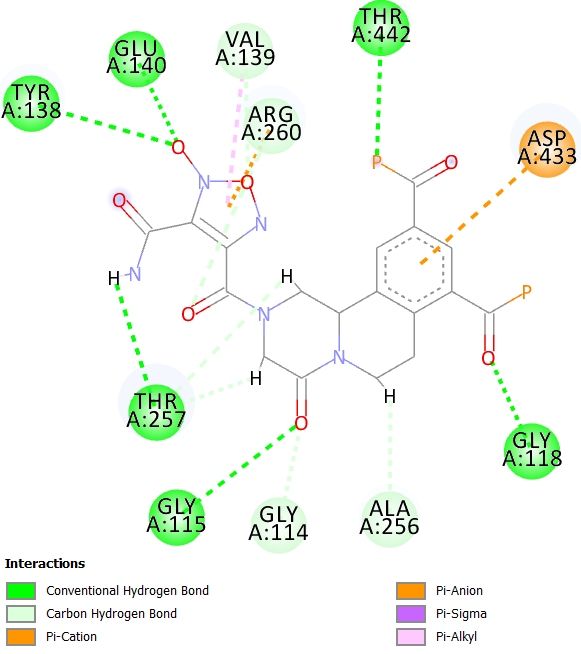

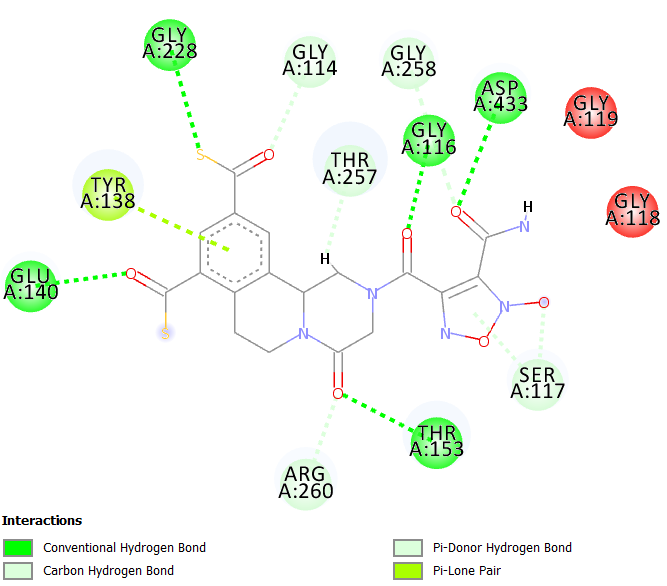

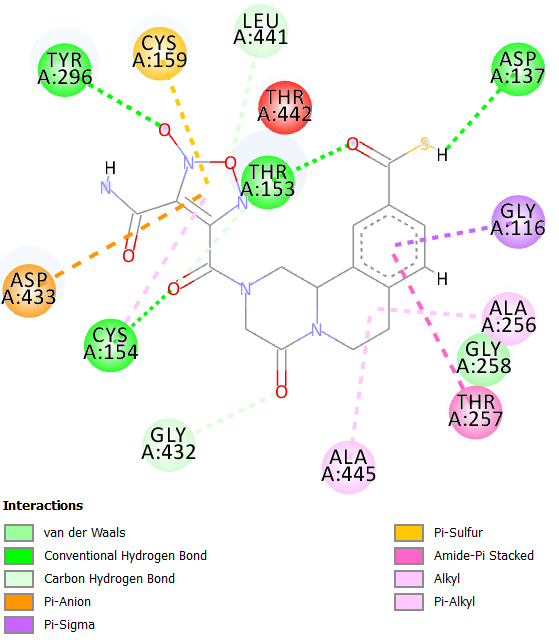

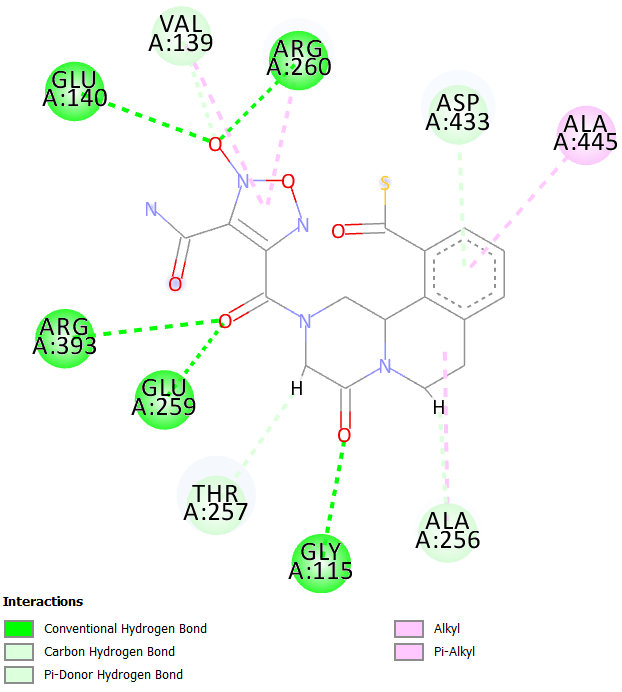


**G**

**H**

**I**

**J**

**K**

**L**

**Figure S1:** 2-Dimentional interactions of designed compounds with 6ZST, (**A**) 6ZST complex with 40a; (**B)** 6ZST complex with 40b; (**C)** 6ZST complex with 40c; (**D)** 6ZST complex with 40d; (**E)** 6ZST complex with 40e; (**F)** 6ZST complex with 40f; (**G)** 6ZST complex with 40g; (**H)** 6ZST complex with 40h; (**I)** 6ZST complex with 40i; (**J)** 6ZST complex with 40j; (**K)** 6ZST complex with 40k; (**L)** 6ZST complex with 40l
